# An extension of Trust and TAM model with TPB in the adoption of digital payment: An empirical study in Vietnam

**DOI:** 10.12688/f1000research.157763.2

**Published:** 2025-03-11

**Authors:** Truong Tuan Linh, Nguyen Thi Thanh Huyen

**Affiliations:** 1 Faculty of Business and Economics, Phenikaa University, Hanoi, Hanoi, 12116, Vietnam

**Keywords:** acceptance behaviors, C-TAM-TPB, digital payment, digital economy, trust-related issues

## Abstract

**Background:**

Digital payment systems are pivotal in the digital economy, relying on the interplay between internet technology and e-vendors. While the Technology Acceptance Model (TAM) and the Theory of Planned Behavior (TPB) have been extensively used to explain technology adoption, the role of trust in financial technology adoption remains underexplored. This study addresses this gap by developing an extended Trust-TAM-TPB model, providing a comprehensive framework to analyze digital payment adoption in emerging markets.

**Methods:**

A quantitative approach was adopted, analyzing survey data from 509 respondents using Structural Equation Modeling (SEM). The extended model examines both technological factors (perceived usefulness, perceived ease of use) and trust-related factors (trust’s influence on behavioral intention via subjective norms, attitude, and perceived behavioral control).

**Results:**

Findings confirm that trust is a significant determinant of digital payment adoption, influencing both perceived usefulness and subjective norms. However, a negative relationship was found between perceived usefulness (PU) and attitude (ATT), suggesting that while users recognize the benefits of digital payments, their attitudes may still be shaped by traditional cash-based habits and security concerns. These insights challenge traditional TAM assumptions and emphasize the importance of trust in driving adoption.

**Conclusions:**

This study contributes to technology acceptance literature by integrating trust into the TAM-TPB framework and highlighting its dual role in shaping both perceived usefulness and behavioral intention. Practically, the findings suggest that policymakers and financial institutions should prioritize trust-building strategies, including fraud prevention measures, financial literacy programs, and transparent transaction policies, to accelerate digital payment adoption in developing economies. These insights are particularly relevant for Vietnam’s Northern mountainous regions, where digital payment penetration remains low.

## 1. Introduction

The transformative power of information technology has reshaped global lifestyles, particularly in communication, the trade of goods and services, and financial transactions. The rise of information and communication technology, along with the increasing online capabilities of devices, has significantly influenced modern life, driving a shift toward greater reliance on machines for decision-making. This transformation is not just a trend but a fundamental shift that is shaping the future of our society ( Susanto et al., 2022). This has led to a dynamic transformation of the global payment system. It is evident in the shift from cash-based transactions to digital payment methods ( Kabir et al., 2017).

Transforming into a digital economy is both an objective and an urgent necessity for Vietnam as it continues to integrate more deeply into the international economy. A key focus of this transformation is the development of digital payments (DPs), which drives advancements in the national population database, e-government, electronic public services, e-commerce, and non-cash payments. The recent rapid and widespread growth of digital payments is a testament to the success of this digital transformation. However, this development also presents challenges that must be addressed with suitable solutions moving forward ( Dao, 2023). As one of the emerging economies in Southeast Asia, Vietnam holds significant potential for the growth of digital payments. In 2021, the total transaction value of digital payments in Vietnam was estimated at $15 billion, with an anticipated annual growth rate of 15.7% through 2025 (PWC Vietnam, 2021).

In recent years, digital payments have rapidly developed in Vietnam. The COVID-19 pandemic has accelerated this trend, with many Vietnamese consumers increasingly opting for digital payment methods. Digital payments provide greater convenience than traditional payment methods, enabling users to complete transactions quickly, from any location, at any time, and reduce costs ( Teng & Khong, 2021; Zhang et al., 2023). The adoption of digital payments is rapidly increasing among individuals, businesses, and public sector organizations ( Kabir et al., 2017).

Several studies have explored the factors that influence the adoption of digital payments. These studies typically utilize well-established frameworks, including the Theory of Reason of Action (TRA) (L. T. B. Diep, 2021), Technology Acceptance Model (TAM) (Davis,1989), the Unified Theory of Acceptance and Use of Technology (UTAUT), or its successor UTAUT2 ( Ramayanti et al., 2024; Venkatesh et al., 2012). Susanto et al. (2022) and Kabir et al. (2017) researched 597 and 223 digital payment articles, respectively, found that TAM theory was the most widely used to analyze factors influencing intent and even the continuity of using digital payments. However, TAM does not include social factors and behavioral control that many studies have shown to influence users’ actual use of new technology significantly ( Taylor & Todd, 1995a). Hence, the researchers proposed a C-TAM-TPB model by combining TPB model (Theory of Planned Behavior) and TAM model ( Chih Chung, 2013; Lee, 2009; Poon et al., 2024; Taylor & Todd, 1995b; Wu & Chen, 2005).

As far as we know, there is limited research on digital payment adoption (DPA) in Vietnam. N. N. Dung et al.(2021) combined UTAUT, TAM, and TPB in their research and indicated that mobility, accessibility, compatibility, convenience, and personal innovation have impacted the intention to use mobile payments. Owning accounts with financial intermediaries positively influenced the use of mobile payments by using the logit regression model ( Son et al., 2020). Based on the positive determinants from previous studies, L. T. B. Diep (2021) found a solid and conclusive relationship between perceived trust, technical protection, perceived security, and e-payment system retention. ECM (Expectation Confirmation Model) and TAM were employed in the study, and the results indicate that trust, in the context of satisfaction, significantly influences the intention of Vietnamese customers to continue using e-wallets ( Thao & Ngoc, 2022).

Digital payments have transformed financial transactions, particularly in the wake of technological advancements and changing consumer behaviors. The increasing reliance on digital payment methods has sparked significant academic interest, particularly regarding the factors influencing user adoption. While the TAM and TPB have been widely applied to explain technology acceptance, emerging research highlights the critical role of trust in financial technology adoption ( Gefen et al., 2003; McKnight et al., 2002). However, limited research has examined how trust integrates with TAM and TPB to shape digital payment adoption, particularly in regions with low digital financial literacy and persistent cash-based economies. This study seeks to address this gap by developing an extended model Trust-TAM-TPB to examine digital payment adoption in Vietnam’s Northern mountainous provinces. This context presents a unique case, as digital payment adoption remains relatively low due to trust concerns, infrastructure limitations, and entrenched consumer habits. By analyzing responses from 509 users through Structural Equation Modeling (SEM), this research aims to contribute both theoretically and practically by providing new insights into the role of trust in shaping digital financial behavior. The study findings offer implications for financial institutions, policymakers, and technology providers aiming to accelerate digital payment adoption in developing markets.

The rest of the paper is structured as follows: Section 2 provides theoretical background. Section 3 outlines the methods. Section 4 presents empirical results. Section 5 focuses on discussing the main results and suggesting some implications. Finally, the authors conclude some limitations and raise potential areas for future research in Section 6.

## 2. Theoretical background

Although digital payment is a specific component of the digital economy, its adoption fundamentally involves the interplay between internet technology and e-vendors in delivering services. The trust and Technology Acceptance Model (TAM) developed by Gefen et al. (2003) has been extensively studied in online shopping contexts. It demonstrates that comprehending both internet technology and trust issues is crucial for understanding behavioral intentions to use online shopping. Therefore, adopting digital payment can be influenced by various potential antecedents, including individual factors, organizational members, and social systems.

Recent studies have highlighted the rapid evolution of digital payment systems, particularly in response to increased digitalization and post-pandemic consumer behavior shifts. Siagian et al. (2022) investigated the role of trust and security concerns in digital payment adoption, emphasizing the interplay between technological ease of use and consumer confidence. Meanwhile, Ranpariya et al. (2023) applied the UTAUT model to analyze the impact of social influence and security perception on mobile payment adoption. Al-Qudah et al. (2024) further expanded on generational differences in digital payment adoption, identifying Generation Z’s preference for contactless transactions driven by perceived convenience and security. Additionally, Giang et al. (2024) examined how consumer trust mediates the adoption of electronic payments in e-commerce settings. These recent insights reinforce the significance of trust, security, and perceived usefulness in digital payment adoption, further justifying this study’s integration of the Trust-TAM-TPB model.

### 2.1 TAM model

***Perceived usefulness***

Perceived usefulness, which reflects an individual’s strong belief in the benefits of technology, is considered a critical factor in enhancing performance ( Davis, 1989; Taylor & Todd, 1995a). Digital payment systems are deemed useful for customers when they offer substantial services. Despite previous unsatisfactory experiences, customers are likely to continue using digital payment methods if they find them beneficial ( Bhattacherjee, 2001; V. Van Diep, 2017). Perceived usefulness is the most frequently utilized independent variable in prior research for assessing people’s readiness to adopt DPs both at individual and organizational levels ( Kabir et al., 2017). Therefore, we propose the following hypothesis:

H _1_:
*Perceived usefulness (PU) positively influences attitudes (ATT) towards adopting digital payments.*

H _9_:
*Perceived usefulness (PU) positively influences user digital payments adoption (DPA).*

***Perceived ease of use***

Perceived ease of use is defined as “the degree to which an individual believes that using a particular system would be free of effort” ( Davis, 1989). Innovative technology systems that are perceived as easier to use and less complex are more likely to gain acceptance and be adopted by users. Digital payment systems are perceived as easy to use when users find them simple to understand, quick to learn, and straightforward to operate. As a result, perceived ease of use is recognized as a critical factor influencing users’ acceptance and adoption of new technology ( V. Van Diep, 2017; Kabir et al., 2017; Tavera-Mesías et al., 2023). Additionally, a more vital perception of ease of use can enhance consumer confidence in the expected benefits of using technological products ( Daragmeh et al., 2021; Doanh et al., 2022). Accordingly, we propose the following hypothesis:

H _2_:
*Perceived ease of use (PEU) positively influences attitudes (ATT) towards adopting digital payments.*

H _3_:
*Perceived ease of use (PEU) positively influences perceived usefulness (PU) towards adopting digital payments.*

### 2.2 TPB model

***2.2.1 Attitude***

Attitude refers to an individual’s favorable or unfavorable feelings about engaging in a particular behavior ( Davis, 1989; Taylor & Todd, 1995a). Additionally, a positive or negative attitude directly impacts the strength of behavioral beliefs regarding the anticipated significant outcomes ( Wu & Chen, 2005). Therefore, it is more likely for customers to take action to use digital payments if they develop a positive opinion about the adoption of a digital payment method. In line with the above argument, we propose the following hypothesis:

H _10_:
*Attitude (ATT) positively influences user digital payments adoption (DPA).*

***2.2.2 Perceived behavioral control***

Perceived behavioral control represents an individual’s perception of the ease or difficulty involved in carrying out a particular behavior. It relates to beliefs about the presence of factors that may either facilitate or obstruct the performance of the behavior ( Ajzen, 1991, 2002; Rachmawati & Rahardi, 2023). In the context of digital payments, perceived behavioral control refers to a consumer’s perception of having the necessary resources, knowledge, and opportunities to adopt digital payment methods. We propose the following hypothesis:

H _11_:
*Perceived behavioral control (PBC) positively influences user digital payments adoption (DPA).*

***2.2.3 Subjective norm***

Subjective norm refers to an individual’s perception of social pressure to either engage in or refrain from a particular behavior ( Ajzen, 1991). In other words, subjective norm relates to an individual’s normative beliefs about the expectations of others ( Rachmawati & Rahardi, 2023; Wu & Chen, 2005). In our study, subjective norm is defined as consumers’ beliefs about the influence that someone important to them may have on their decision to adopt digital payment methods. Based on this, we propose the following hypothesis:

H _12_:
*Subjective norm (SN) positively influences user digital payments adoption (DPA).*

### 2.3 Trust

**Figure 1.**
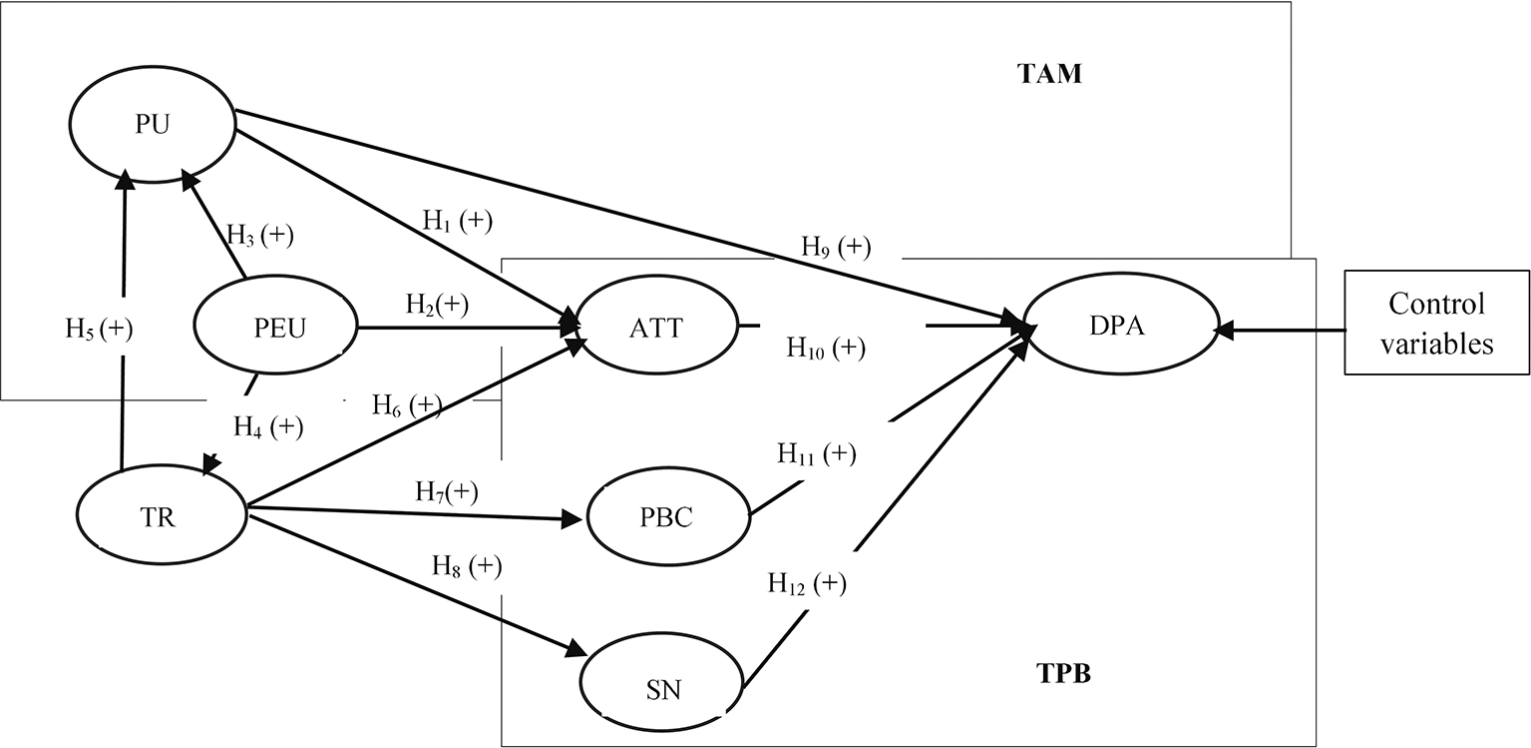
Conceptual framework.

Trust is the confidence one party has in the intentions and actions of the other party ( Aldaabseh & Aljarah, 2021; Siagian et al., 2022). Trust often encompasses three key dimensions: ability, integrity, and benevolence ( Benamati et al., 2010). Ability refers to the knowledge and skills required of digital payment service providers to fulfill their tasks effectively. Integrity signifies that these providers consistently keep their promises, while benevolence indicates that they genuinely care about the users’ interests, not just their own. Trust will be a crucial potential influencer in examining the adoption of digital payments. In exploring the factors influencing digital payment adoption, we refer to the relationships depicted in Figure 1, which presents the conceptual framework guiding our analysis. This framework emphasizes the interplay between technological acceptance and trust

***2.3.1 Trust and TAM relationship***

The relationship between PU, PEU, and trust has been widely discussed in the literature ( Gefen, 2004; Gefen et al., 2003; Pavlou, 2003). Wu & Chen (2005) followed Gefen et al. (2003) suggest a model of trust and TAM for the initial adoption of online tax. It shows that trust is an antecedent of PU, and PEU is an antecedent of trust.

In line with social cognitive theory, perceived ease of use (PEU) can generally be argued to positively impact a person’s favorable expectations regarding accepting innovative technology ( Bandura, 1986). This is because cognition-based trust is primarily founded on initial impressions of behavior. In the context of online services, PEU represents the initial feeling or expectation that influences further online transactions ( Wu & Chen, 2005). PEU is hypothesized to positively influence trust because it helps foster a favorable impression of e-vendors during the initial adoption of online services. This positive impression can make customers more willing to invest in and commit to the buyer-seller relationship ( Gefen et al., 2003; Wu & Chen, 2005). In line with the above argument, we propose the following hypothesis:

H _4_:
*Perceived ease of use (PEU) positively influences Trust (TR) towards adopting digital payments.*

Trust is a key determinant of perceived usefulness, particularly in the digital payments environment, as it assures consumers that they will experience the expected benefits from service providers. In addition, trust is recognized to positively influence PU because it allows consumers to feel comfortable being vulnerable with e-vendors, ensuring they receive the expected value from their interactions and services ( Pavlou, 2003). When consumers initially trust their e-vendors and perceive that adopting an online tax will enhance their job performance, they are more likely to believe that the online tax is beneficial ( Wu & Chen, 2005). Hence, we propose the following hypothesis:

H _5_:
*Trust (TR) positively influences perceived usefulness (PU) towards adopting digital payments.*

***2.3.2 Trust and TPB relationship***

The relationship between trust and TPB can be explored from various perspectives. Trust is hypothesized as a common antecedent influencing attitude, perceived behavioral control, and subjective norm.

Trust in an e-vendor is considered a significant behavioral belief that directly influences a customer’s attitude toward purchasing behavior. When an e-vendor is perceived as trustworthy, it increases the likelihood that the consumer will benefit from and avoid potential risks associated with adopting online services ( Pavlou, 2003). Many previous studies have shown a positive relationship between trust and attitude ( Agag & El-Masry, 2016; Chawla & Joshi, 2019; Wu & Chen, 2005). Trust is clearly a crucial factor influencing attitudes toward adopting digital payment behavior. Thus, the following hypothesis is proposed:

H _6_:
*Trust (TR) positively influences attitude (ATT) towards adopting digital payments.*

Trust can strengthen perceived behavioral control in online transactions by making the interactions between customers and e-vendors more predictable and consistent ( Pavlou, 2003). Trust impacts perceived behavioral control by bolstering self-efficacy and fostering conditions that facilitate successful interactions ( Wu & Chen, 2005). When customers trust a digital payment provider that meets their expectations, this trust is likely to enhance their perceived behavioral control over digital payment transactions. Based on the preceding arguments, the hypothesis can be stated as follows:

H _7_:
*Trust (TR) positively influences perceived behavioral control (PBC) toward adopting digital payments.*

Peer and superior influences shape users’ subjective norms concerning IT usage. Consequently, it can be inferred that trust in peers and superiors concerning their beliefs about IT usage should play a significant role in shaping subjective norms ( Taylor & Todd, 1995a). Similarly, trust in e-vendors, particularly regarding their reputation, brand name, and service quality, may positively influence subjective norms related to online transaction behavior ( Wu & Chen, 2005). Therefore, whether trust exerts direct or indirect influences on subjective norms remains a crucial antecedent of subjective norms in digital payments. Hence, we propose the following hypothesis:

H _8_:
*Trust (TR) positively influences subjective norms (SN) toward adopting digital payments.*

## 3. Methods

### 3.1 Sample profile

This study utilized both quantitative and qualitative research across four phases to explore the factors influencing digital payment adoption in Vietnam’s northern mountainous region. The survey scales were developed based on theoretical frameworks and relevant literature, with input from experts in fintech and e-commerce to ensure content validity. A pilot test with 100 participants from Thai Nguyen and Cao Bang helped refine the questionnaire. The primary survey was conducted in Lang Son and Cao Bang provinces from December 2023 to January 2024. This study employed a multi-stage stratified sampling approach to ensure diverse representation across different demographic segments. The research was conducted in Lang Son and Cao Bang provinces, which were selected due to their relatively low digital payment adoption rates. In the first stage, we identified key districts within each province where digital payments were increasingly promoted. In the second stage, a random sampling technique was used to select individual respondents who had prior exposure to digital payments. Eligibility criteria included: (1) individuals aged 18 and above, (2) those who had engaged in at least one digital transaction in the past six months. This approach ensured a mix of urban and rural respondents, capturing diverse perspectives on digital payment adoption. These provinces were chosen to explore the factors limiting digital payment adoption, particularly in rural areas, as part of a broader investigation into the challenges of digital payment adoption in the northern mountainous region of Vietnam.

Following the sample size guidelines from Hair et al. (2019), we aimed for at least 140 participants, with the final sample comprising 800 respondents evenly split between the two provinces. The sample size of 509 valid responses was determined based on guidelines for Structural Equation Modeling (SEM). Hair et al. (2019) recommend a minimum of 200 cases for SEM analysis, with a ratio of 10:1 per observed variable. Given that our model includes multiple constructs, a sample exceeding 500 was targeted to enhance statistical power and model stability. The sample size follows recommendations from Kline (2023), which suggest that larger samples improve the robustness of SEM path estimates. This ensures that factor loadings and path coefficients remain stable, reducing the risk of sampling bias. Participants were informed about the study and provided consent before the interviews. Each respondent received a small gift valued at approximately $2 to encourage participation. Of the initial surveys, 509 were valid, yielding a 63.63% response rate. Participants provided personal information, including their name, address, age, education, income, and occupation, which interviewers recorded. The interviewees reviewed their responses and confirmed their accuracy by signing the survey form. Afterward, they signed a summary information table and received a gift from the project.

To ensure the accuracy and reliability of the survey instrument, a three-stage validation process was conducted before data collection. First, the questionnaire was developed based on established scales from previous studies on Technology Acceptance Model (TAM), Theory of Planned Behavior (TPB), and Trust in Digital Payments ( Ajzen, 1991; Davis, 1989; Gefen et al., 2003). To assess content validity, the survey items were reviewed by three fintech experts and two academic researchers specializing in technology adoption and digital payments. Their feedback helped refine the wording and ensure the constructs accurately captured user perceptions in the Vietnamese context. Second, a pilot study was conducted with 100 participants from Thai Nguyen and Cao Bang to evaluate the clarity and comprehensibility of the questionnaire. Participants were asked to highlight any ambiguous or difficult-to-understand questions. Based on their feedback, minor wording adjustments were made to improve clarity without altering the intended meaning of the items. Finally, statistical validation of the instrument was planned before hypothesis testing. Specifically, Exploratory Factor Analysis (EFA) and Confirmatory Factor Analysis (CFA) would be employed to assess construct validity, while Cronbach’s alpha, Composite Reliability (CR), and Average Variance Extracted (AVE) would be used to evaluate internal consistency and reliability. The results of these tests are reported in the Results section. By implementing this rigorous validation process before the main data collection, the study ensured that the measurement instrument was robust and suitable for examining the proposed research model.

### 3.2 Method estimation

Structural Equation Modeling (SEM) is a second-generation statistical analysis technique designed to examine multidimensional relationships between multiple variables within a model ( Anderson et al., 2004). SEM has been widely utilized across various fields, including sociology ( Lavee, 1988; Lorence & Mortimer, 1985), psychology ( Anderson et al., 2004), and management ( Tharenou et al., 1994).

The complex theoretical model created using this method is generally associated with the data collected for validation purposes. This association is known as model-data fit. Any theoretical model can be evaluated using available empirical data for this type of fit. SEM is recognized as an extensive sample method, usually necessitating a minimum sample size of 200 ( Dash & Paul, 2021; Hayes et al., 2017). There are two primary models: the path analysis and the measurement model. While some advanced models, such as multilevel and growth models, are also considered, this study will focus on i) the measurement model and ii) path analysis.

Before examining the path analysis among latent variables (also called factors or constructs), we first assess the measurement of these unobserved variables. Since these variables are not directly observable, they are represented through a set of measured variables from which the latent constructs are derived. Each latent variable is quantified using observed indicators tested for reliability and validity. SEM uses Confirmatory Factor Analysis (CFA) to evaluate the measurement model. In this case, the model fit is assessed to validate the measurement model. Once the model fit is confirmed, path models among the latent variables are evaluated ( Hair et al., 2019; Hayes et al., 2017). In our research, before employing SEM, we followed the recommendations of Doanh et al. (2024) and Huy et al. (2024) by conducting exploratory factor analysis (EFA) to identify latent variables. EFA aims to uncover the latent constructs underlying a set of observed variables. EFA is used when the research goal is to determine the nature and number of common factors among these variables ( Omura et al., 2022). Subsequently, we performed confirmatory factor analysis (CFA) by examining factor loadings, composite reliability (CR) indexes ( Joreskog et al., 1971), and average variance extracted (AVE). While not a parameter estimate, AVE helps evaluate how much of the variance in indicators is explained by the latent factor. Calculated using factor loadings and residual error variances for a latent factor, AVE values of 0.50 or higher are generally acceptable. Composite Reliability (CR), like AVE, is not a parameter estimate but is valuable for interpreting the CFA model. Generally, CR values of 0.70 or higher are considered acceptable; however, values between 0.60 and 0.70 may indicate questionable reliability but are not necessarily unacceptable ( Fornell & Larcker, 1981; Joreskog et al., 1971). This methodology, commonly referenced in the literature, rigorously assesses our research constructs and ensures the validity and reliability of our findings.

The path model is a form of multiple regression model estimated simultaneously, illustrating mediation, moderation, and interaction effects among variables. It defines the structural relationships between latent variables based on their associations with observed indicators (measured variables). Once the measurement models of latent constructs are validated through CFA, these paths can be interpreted as causal or covariance-based. It can assess the unidimensionality, validity, and reliability of an unobserved latent construct (factor) ( Dash & Paul, 2021; Hair et al., 2019).

Some of the fit indices in the SEM model are used to test and compare models, such as Chi-square, Comparative fit index (CFI), Tucker–Lewis Index (TLI), and Root mean squared error of approximation (RMSEA). Overall model fit is evaluated using the chi-square statistic, which reflects the discrepancy between the sample data and the model’s specified covariance matrices. This statistic is widely regarded as an indicator of model fit quality, with a non-significant value at the 0.05 level being preferable. Additionally, the chi-square to degrees of freedom ratio (CMIN/df ) provides a more straightforward assessment, with a value of 3 or less (sometimes up to 5) typically indicating a good fit. The Comparative Fit Index (CFI) is an incremental fit index that compares the focal model to a baseline model, often called the null or independent model. CFI tends to be less affected by sample size than the chi-square test. A CFI value of 0.95 or above typically signifies a good fit to the data, although some may consider a threshold of 0.90 acceptable. The Tucker-Lewis Index (TLI) is a comparative or incremental fit index. Generally, a TLI value of 0.95 or higher indicates a good model fit to the data, though some guidelines permit a lower cutoff of 0.90. The Root Mean Square Error of Approximation (RMSEA) is an absolute fit index that penalizes model complexity, favoring more parsimonious models. Generally, an RMSEA value of 0.06 or lower indicates a good fit to the data, although some guidelines may relax this threshold to 0.08 or even 0.10 ( Bagozzi & Yi, 1988; Dash & Paul, 2021).

We selected SEM as the analysis method for this study due to its robustness in testing theories, as highlighted by Steenkamp & Baumgartner (2000). This makes SEM particularly suited to our research focus. Our research model encompasses multidimensional constructs that are not directly observable but are measured through observable indicators. Thus, SEM, which emphasizes construct operationalization ( Bagozzi, 1994), is an appropriate and practical approach for our investigation. Furthermore, our application of SEM is consistent with prior research ( Thongsri et al., 2019).

Followed Fan et al. (2016); Kline (2018), we deploy SEM, which consists of five main steps: model specification, identification, parameter estimation, model evaluation, and model modification. The model specification defines hypothesized relationships among variables based on prior knowledge, while model identification checks if the model is over-identified, just-identified, or under-identified, as coefficients can only be estimated in just-identified or over-identified models. Parameter estimation then calculates these coefficients. The model evaluation assesses how well the model fits using quantitative indices to measure the overall goodness of fit. If necessary, model modification adjusts the model to improve fit, often in a post hoc fashion. Finally, validation enhances the model’s reliability and stability. In our study, SEM analysis was conducted using JASP ( https://jasp-stats.org/).

### 3.3 Data description

Table 1 presents the demographic features of the respondents to the survey. According to the study’s gender review, 42.6% are male, and 57.4% are female. Most respondents are between the ages of 31 and 40 (229 individuals, accounting for 45%), followed by the age range of 41 to 50 years (143 individuals, accounting for 28.1%). In terms of culture, the majority are ethnic minorities such as Tay and Nung, accounting for 78.2%, while the remaining belong to the Kinh and other groups. Regarding educational level, most interviewers had completed college or university degrees, accounting for 56.7%, followed by those who graduated high school (24.6%). Those with education levels below secondary school and secondary school constituted a small proportion (10.4%). Most families have 4 to 5 members, accounting for 61.1%. The average monthly income of people is meager; the number with income equal to or greater than 5 million VND/month accounts for only 29.1%, and the rest all have income below 5 million. One of the reasons is that their primary income comes from their profession, in which data shows that 44.6% are purely farmers, the remaining 55.4% are workers working away from home, and commune-level civil servants.

**Table 1.** The demographic information of the participants.

Variables	Category	Frequency	Percent
Gender	Male	217	42.6
	Female	292	57.4
Age	Under 20	4	0.8
	21 – 30	87	17.1
	31 – 40	229	45.0
	41 – 50	143	28.1
	Over 50	46	9.0
Culture	Kinh	111	21.8
	Others	398	78.2
Educational level	None	13	2.6
	Primary school	36	7.1
	Secondary school	17	3.3
	High school	125	24.6
	Vocational school	29	5.7
	College, University	241	47.3
	Master	48	9.4
Income (Million VND/month)	Under 1	74	14.5
	2	84	16.5
	3	80	15.7
	4	123	24.2
	≥ 5	148	29.1
Household size (number of people in family)	1	4	0.8
	2	26	5.1
	3	66	13.0
	4	184	36.1
	5	127	25.0
	6	78	15.3
	> 6	24	4.70
Job	Farmer	227	44.6
	Others	282	55.4

**Source:** Authors.

## 4. Results

### 4.1 Measurement model

This study follows a two-step SEM approach as recommended by Hair et al. (2019). In the first step, the measurement model was assessed using Confirmatory Factor Analysis (CFA) to ensure construct validity, reliability, and overall model fit. Only after the measurement model demonstrated an acceptable fit was the structural model estimated. The second step involved testing the structural model, where maximum likelihood estimation (MLE) was applied to examine the relationships among latent variables. MLE was selected due to its robustness in handling non-normality and its effectiveness when the sample size exceeds 200, as supported by Kline (2018).

Before proceeding with SEM model testing to evaluate the hypotheses, we conducted EFA and CFA to assess the variables’ construct and confirm the reliability and validity of the measurement model. Table 2 shows that the total Cronbach’s alpha is 0.938 greater than 0.6, and all observed variables have Cronbach’s alpha coefficients ≥ 0.935. The results also indicate that seven variables were extracted from 30 observed factors; the eigenvalue value is 1.053 (>1) with a variance of 71.62%. The results of the EFA in Table 2 show that the factor loadings of the items ranged from 0.661 to 0.853, and the construct of items is consistent with the literature. The test coefficient KMO = 0.916 satisfies the 0.5 < KMO< 1, showing that the exploratory factor analysis is appropriate for our data. The Chi-square statistic of the Bartlett test reached 9630.515, with the p-value = 0.000, showing that the data is suitable and reliable for performing the EFA method.

**Table 2.** The results of Cronbach’s alpha and Exploratory Factor Analysis.

Variable	Cronbach’s alpha (CA)	Factor1	Factor2	Factor3	Factor4	Factor5	Factor6	Factor7
PU1	0.937							0.796
PU2	0.937							0.821
PU3	0.936							0.772
PEU1	0.937						0.745	
PEU2	0.937						0.749	
PEU3	0.937						0.805	
PEU4	0.937						0.788	
TR1	0.935		0.741					
TR2	0.935		0.780					
TR3	0.936		0.829					
TR4	0.936		0.853					
TR5	0.937		0.813					
ATT1	0.937				0.685			
ATT2	0.936				0.767			
ATT3	0.936				0.799			
ATT4	0.935				0.716			
SN1	0.935	0.725						
SN2	0.935	0.733						
SN3	0.936	0.734						
SN4	0.935	0.758						
SN5	0.935	0.726						
SN6	0.935	0.661						
PBC1	0.935					0.682		
PBC2	0.935					0.717		
PBC3	0.936					0.758		
PBC4	0.935					0.736		
DPA1	0.936			0.721				
DPA2	0.936			0.837				
DPA3	0.936			0.809				
DPA4	0.936			0.766				

KMO = 0.916 the Bartlett test χ ^2^ = 9630.515, p-value = 0.000.

**Source:** Authors.

CFA allows for testing the validity and accuracy of specific models constructed based on data and theoretical foundations. We assess the goodness-of-fit of the measurement model using various tests, including the Chi-square test (χ ^2^), Comparative fit index (CFI), Tucker–Lewis Index (TLI), and Root mean squared error of approximation (RMSEA), composite reliability (CR) and the average variance extracted (AVE). As shown in Table 3, the composite reliability (CR) of latent variables exceeds 0.836, and the AVE values range from 0.560 to 0.687. In addition, several fit indices were analyzed. The chi-square statistic (χ ^2^/df = 2.486) was within the acceptable threshold of less than 3, indicating a good fit ( Hair et al., 2019). The CFI = 0.943 and TLI = 0.931 were both above the recommended threshold of 0.90, confirming that the model exhibited strong comparative and incremental fit. Additionally, the RMSEA = 0.05 was below the upper limit of 0.08, signifying a satisfactory absolute fit. The SRMR = 0.03 was well below the 0.08 cutoff, further reinforcing the model’s robustness. Collectively, these indices demonstrate that the proposed model achieves an adequate fit, allowing for meaningful interpretation of the structural relationships.

**Table 3.** The results of confirmatory factor analysis.

Items	Coef.	OIM Std. Err	P-value	Composite Reliability (CR)	Average Variance Extracted (AVE)
**Perceived Usefulness- PU**				**0.840**	**0.637**
PU1	0.757	0.024	0.000		
PU2	0.805	0.022	0.000		
PU3	0.829	0.021	0.000		
**Perceived Ease of Use-PEU **				**0.836**	**0.560**
PEU1	0.735	0.041	0.000		
PEU2	0.751	0.037	0.000		
PEU3	0.895	0.032	0.000		
PEU4	0.761	0.034	0.000		
**Trust- TR**				**0.913**	**0.679**
TR1	0.913	0.048	0.000		
TR2	0.885	0.028	0.000		
TR3	0.793	0.029	0.000		
TR4	0.784	0.043	0.000		
TR5	0.753	0.051	0.000		
**Attitude- ATT**				**0.864**	**0.614**
ATT1	0.754	0.024	0.000		
ATT2	0.802	0.021	0.000		
ATT3	0.769	0.024	0.000		
ATT4	0.777	0.023	0.000		
**Subjective Norm- SN**				**0.860**	**0.606**
SN1	0.797	0.022	0.000		
SN2	0.774	0.021	0.000		
SN3	0.711	0.024	0.000		
SN4	0.817	0.019	0.000		
SN5	0.800	0.018	0.000		
SN6	0.763	0.023	0.000		
**Perceived Behavioral Control- PBC**				**0.865**	**0.615**
PBC1	0.783	0.026	0.000		
PBC2	0.794	0.026	0.000		
PBC3	0.777	0.023	0.000		
PBC4	0.808	0.022	0.000		
**Digital Payment Adoption-DPA **				**0.897**	**0.687**
DPA1	0.899	0.040	0.000		
DPA2	0.853	0.017	0.000		
DPA3	0.828	0.019	0.000		
DPA4	0.825	0.018	0.000		

LR test of model vs. saturated: chi ^2^ (361) = 897.54 Prob > chi ^2^ = 0.0000.

**Source:** Authors.

### 4.3 Structural equation model

**Figure 2.**
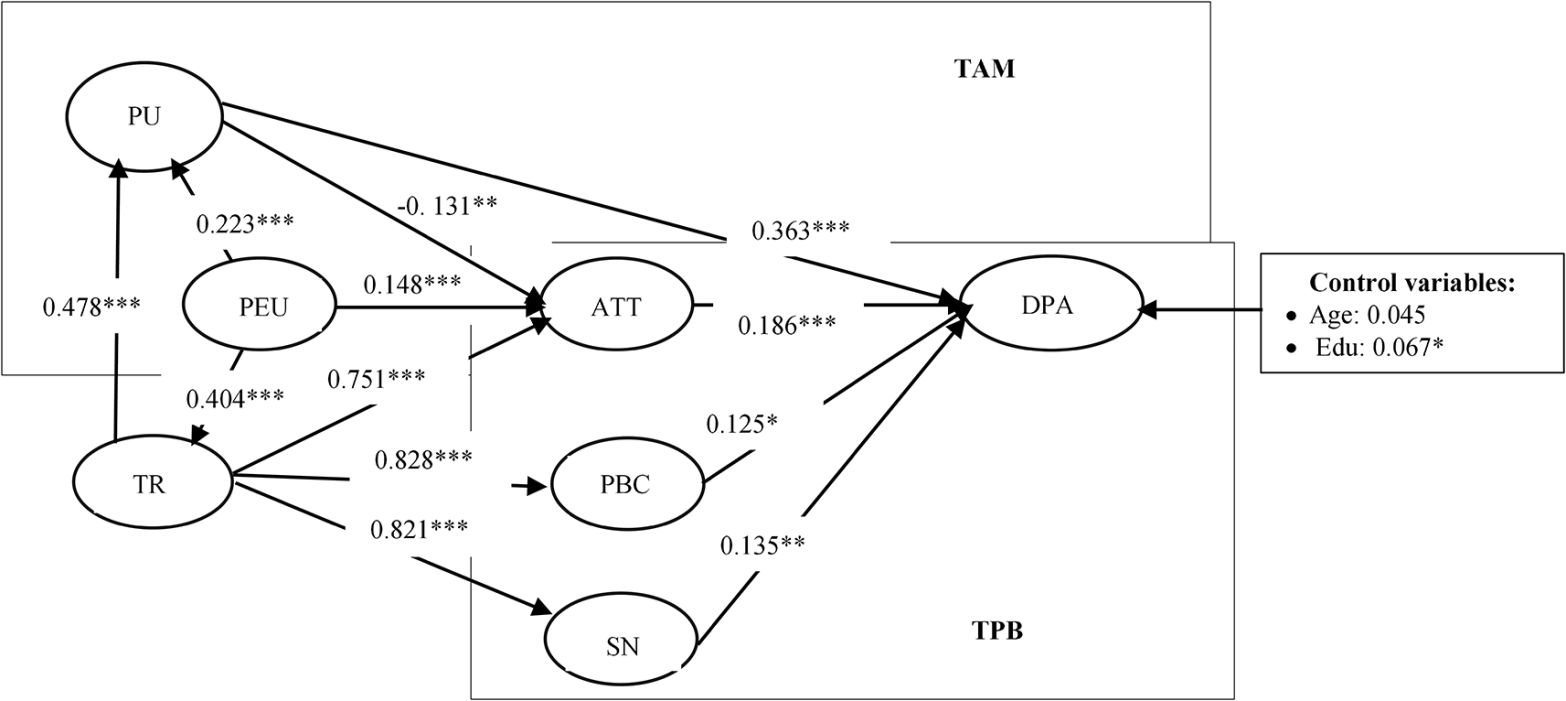
The results of the structural equation model.

Table 4 and Figure 2 present the model’s estimation results using the SEM method. The assessment revealed favorable fit index values: χ ^2^/df = 3.014, CFI = 0.909; TLI = 0.895, RMSEA = 0.06, SRMR= 0.05. These results indicate that the empirical findings are reliable and valid.

**Table 4.** The results of the structural equation model.

Variables	Coef.	Std. Err	z- value	p-value
**Dependent variable: DPA**				
PU	0.363	0.045	8.10	0.000
ATT	0.186	0.062	3.03	0.002
SN	0.135	0.061	2.20	0.028
PBC	0.125	0.066	1.90	0.057
Age	0.045	0.365	1.22	0.222
Edu	0.067	0.037	1.81	0.070
**Dependent variable: PU**				
TR	0.478	0.048	9.94	0.000
PEU	0.223	0.050	4.45	0.000
**Dependent variable: ATT**				
PU	-0. 131	0.058	-2.26	0.024
TR	0.751	0.052	14.41	0.000
PEU	0.148	0.048	3.06	0.002
**Dependent variable: TR**				
PEU	0.404	0.046	8.73	0.000
**Dependent variable: PBC**				
TR	0.828	0.025	33.34	0.000
**Dependent variable: SN**				
TR	0.820	0.024	34.24	0.000

LR test of model vs. saturated: chi ^2^ (428) = 1289.86Prob > chi ^2^ = 0.0000.

**Source:** Authors.

As a result, all hypotheses are positive and statistically significant at the 1% level. It means that all hypotheses are supported.

The results from the structural model estimation support most of the hypothesized relationships, confirming the theoretical foundations of the Trust-TAM-TPB model in the context of digital payment adoption. Notably, perceived ease of use (PEU) was found to have a significant positive impact on trust (β = 0.404, p < 0.001). This finding aligns with previous research ( Gefen et al., 2003), which suggests that when users perceive a digital payment system as easy to use, they are more likely to develop confidence in its reliability and security. Additionally, trust was shown to significantly enhance perceived usefulness (PU) (β = 0.478, p < 0.001), reinforcing the notion that users find digital payment systems more beneficial when they have confidence in their security and operational effectiveness.

A key theoretical confirmation from the findings is the strong influence of attitude (ATT) on digital payment adoption (DPA) (β = 0.186, p = 0.002). This result supports Hypothesis H _10_ and aligns with the Theory of Planned Behavior (TPB), where a positive attitude toward a technology significantly increases the likelihood of its adoption. Similarly, subjective norms (SN) and perceived behavioral control (PBC) were also found to be strong predictors of adoption, further reinforcing the relevance of TPB in digital payment contexts. However, an unexpected finding emerged regarding the relationship between perceived usefulness (PU) and attitude (ATT) (β = -0.131, p = 0.024). Contrary to previous research based on TAM ( Wu & Chen, 2005), this result suggests that users in the studied regions may not necessarily develop a positive attitude toward digital payments even when they acknowledge their usefulness. A potential explanation is that long-standing cash-based habits in Vietnam’s Northern mountainous areas create resistance to shifting to digital transactions, despite recognizing their benefits. This insight suggests that beyond promoting usefulness, digital payment providers should emphasize trust-building strategies and behavioral incentives to encourage attitudinal change and subsequent adoption.

Overall, the findings validate the core structure of the Trust-TAM-TPB model while revealing context-specific nuances that should be considered in digital payment adoption strategies. The integration of trust within the model highlights its essential role in shaping perceived usefulness and behavioral intentions, offering practical insights for financial institutions and policymakers aiming to increase digital payment penetration in emerging markets.

### 4.4 Additional analysis

It is crucial to emphasize the importance of addressing the Skewness-Kurtosis test. To tackle this issue, we consulted the studies by Barnes et al. (2001) and Vieira (2011). Additionally, following the recommendation of Diamantopoulos & Siguaw (2000) we conducted a model cross-validation analysis.

Our analysis used multivariate normality tests, such as the Mardia mSkewness and Mardia mKurtosis tests, to evaluate our model’s performance. The findings reveal that our model satisfied the criteria set by both the mKurtosis and mKurtosis tests. This means that our research model is appropriate for maximum likelihood (ML) estimation and demonstrates a good fit.

$$\begin{array}{lll} \text{Mardia mSkewness}=171.5646 & {\text{chi}}^{2}(4960)=14645.737 & \text{Prob}>{\text{chi}}^{2}=0.0000\\ \text{Mardia mKurtosis}=1303.712 & {\text{chi}}^{2}(1)=7829.700 & \text{Prob}>{\text{chi}}^{2}=0.0000 \end{array}$$

## 5. Discussion

### 5.1 Discussion results

This study aims to utilize an extended version of the Trust and TAM model, incorporating the TPB, to better understand the acceptance behavior toward adopting digital payments in the Northern mountainous area of Vietnam. The empirical results from the SEM model reveal that the standardized path coefficients are all positively significant except for the path from PU to ATT.

***5.1.1 TAM model***

Perceived usefulness (PU) negatively influences attitudes (ATT) towards adopting digital payments at the 5% level, as supported by the standard regression coefficient value of -0.131. Therefore, the hypothesis H _1_ has been rejected. This result contrasts previous studies ( Ariffin et al., 2021; Chawla & Joshi, 2019; Mabkhot et al., 2023; Ranpariya & Joshi, 2024; Wu & Chen, 2005). This indicates that users do not feel digital payments are useful. This may come from their daily habits of using cash instead of digital payment methods. Therefore, it is essential to keep expanding the spread of information and communication regarding the practical advantages of digital and cashless payments. Additionally, implementing safeguards and strict measures to prevent fraud and property theft on online platforms is equally important.

Our findings align with prior literature on technology adoption and trust, reinforcing the idea that perceived ease of use (PEU) and trust (TR) are critical for overcoming digital payment resistance ( Gefen et al., 2003; McKnight et al., 2002). The negative PU → ATT relationship observed in this study suggests that usefulness alone is not sufficient in encouraging adoption, especially in economies with entrenched cash-based behaviors. This aligns with knowledge-sharing theories, where social influence and trust networks play a crucial role in facilitating the adoption of new technologies ( Chow & Chan, 2008; Wang & Noe, 2010).

Our results also show the positive influence of perceived usefulness (PU) at the 1% significance level on DPA, thereby supporting H _9_. Conclusively, the results obtained are supported by the results of previous studies ( Aji et al., 2020; Chawla & Joshi, 2019; Nguyen & Ao, 2022; Ranpariya & Joshi, 2024; Siagian et al., 2022). This means that when consumers achieve significant benefits from digital payment systems, they will trust DP more and continue to use it. Therefore, service providers should focus on further developing and improving the quality of DP to increase customers’ perception of its usefulness. This will help increase the number of users adopting DPs.

The findings of H _2_ align with previous studies, showing that perceived usefulness positively and significantly influences users’ attitudes toward using digital payment ( Ariffin et al., 2021; Mabkhot et al., 2023); however, in contrary to Chawla & Joshi (2019). This study’s findings indicate that consumers found it easy to conduct payment transactions using a digital payment system. The system not only streamlined their payment processing but also enhanced their transaction efficiency, leading to a positive attitude toward adopting digital payments.

Furthermore, the finding of H _3_ is consistent with previous research, which demonstrated that perceived ease of use positively and significantly influences perceived usefulness at the 1% level ( Aji et al., 2020; Chawla & Joshi, 2019; Ranpariya & Joshi, 2024; Siagian et al., 2022; Wu & Chen, 2005). It proved that perceived ease of use increases the perceived usefulness of adopting digital payments. Therefore, service providers should focus on further developing and improving their payment system to be simpler to understand, quick to learn, and straightforward to operate digital payment services.

***5.1.2 TPB model***

Attitude (ATT) was also determined to impact DPA at the 1% significance level positively; hence, hypothesis H _10_ is supported. It means that customers have a positive opinion about adopting digital payment methods. This study is also in accordance with the results of research by Ariffin & Lim (2020); Ayudya & Wibowo (2018); Chawla & Joshi (2019); Ranpariya & Joshi (2024).

Perceived behavioral control (PBC) is supposed to positively affect the use of DP at the 1% level. This hypothesis is supported by the value of the standard regression coefficient (0.178). Therefore, the acceptance of hypothesis H _11_ has been verified. This finding reveals that consumers nowadays have enough resources, knowledge, and opportunities to adopt a digital payment method. This result was consistent with the results of Ariffin et al. (2021); Ariffin & Lim (2020); Ayudya & Wibowo (2018); Mabkhot et al. (2023).

Similarly, subjective norm (SN) also positively impacts users’ adoption of digital payments at the 1% significance level. Hence, hypothesis H _12_ is supported. The result aligns with the viewpoints of Aji et al. (2020); Ariffin et al. (2021); Jusoh & Jing (2019) but not consistent with Ayudya & Wibowo (2018). This insight demonstrates that people’s willingness to adopt digital payments as a new mode of transaction is significantly influenced by their peers’ opinions and behaviors. Reliable information from trusted sources like relatives, neighbors, or friends boosts consumers’ trust in digital payment methods, leading to a stronger intention to adopt them. Findings related to subjective norms (SN) further support the knowledge-sharing perspective, as social influence plays a key role in shaping user trust in digital payments. Reliable information from trusted sources like friends, family, or communities boosts confidence, reinforcing prior research that suggests trust-based peer recommendations are essential for technology adoption ( Bock et al., 2005; Lin, 2007).

***5.1.3 Trust and TAM relationship***

Perceived ease of use (PEU) is found to positively and significantly influence trust (TR) towards adopting digital payments, and hypothesis H _4_ is supported. The result in light of the previous studies, which also showed the importance of PEU in enhancing user trust ( Chawla & Joshi, 2019; Gefen et al., 2003; Wu & Chen, 2005). It reveals that consumers had a favorable impression of e-vendors and trust when using digital payment transactions.

Moreover, trust (TR) positively impacts perceived usefulness (PU). This relationship was proved by the value of the standard regression coefficient of 0.478 at the 1% significant level. Hence, the hypothesis H _5_ is supported. It confirmed that consumers feel comfortable being vulnerable with digital payment service providers and can receive the expected value from their actions and services. Then, they will adopt more and more digital payment methods for their daily buy and sell activities. Previous studies support this result ( Gefen et al., 2003; Wu & Chen, 2005).

***5.1.4 Trust and TPB relationship***

Our research findings corroborate those of ( Ariffin & Lim, 2020; Wu & Chen, 2005), who similarly suggested that trust (TR) positively influences attitude (ATT) towards adopting digital payments, with a coefficient of 0.751 and a p-value of 0.000. Based on these findings, the hypothesis H _6_ is confirmed. This supports the previous findings on trust and attitude, showing that when consumers believe in the services and information of e-vendors, they will increase the likelihood of adopting digital payment methods.

The positive significance of H _7_ proves that trust (TR) boosts perceived behavioral control (PBC) in online transactions by making interactions between customers and e-vendors more stable and predictable. This result is consistent with the studies of Ariffin & Lim (2020); Wu & Chen (2005). It confirms that when customers trust a digital payment provider that fulfills their expectations, this trust will likely increase their perceived control over digital payment transactions.

Notably, the results have highlighted the significant role of trust (TR) in influencing subjective norm (SN) toward adopting digital payments. This trust has a positive impact on subjective norm, with a coefficient of 0.821 and a p-value of 0.000. This result is not in line with research conducted by Ariffin & Lim (2020) but consistent with Wu & Chen (2005). It shows that customers in this region had trust in e-vendors, particularly in terms of their reputation, brand recognition, and service quality, which may positively affect subjective norm related to digital payment transaction behavior.

Among the control variables, the results show that age is not statistically significant, suggesting that there is no difference in the adoption of DPs based on the respondents’ age. Education is found to have a positive significance with DPA at a 10% level and in light with Cao et al. (2018). It proves that users with higher education levels are more likely to use DP systems to transfer money, shop, and pay for utility services.

This study extends the literature by showing that trust acts as a bridge between technological perceptions (TAM) and behavioral intentions (TPB), further reinforcing the interconnected role of technology and trust in financial decision-making. The dual role of trust in shaping both perceived usefulness (PU) and behavioral control aligns with broader fintech research, emphasizing that trust is a prerequisite for digital financial engagement ( Nguyen & Ao, 2022; Siagian et al., 2022).

### 5.2 Theoretical contributions

The previous body of literature concerning intentions to adopt digital payments has primarily relied on established theories such as TRA, TAM, TPB, UTAUT, and UTATU2 ( Kabir et al., 2017; Susanto et al., 2022). In line with this existing research, the findings of this study provide significant theoretical contributions by extending the TAM and TPB with Trust in the context of digital payment adoption. While previous studies have separately examined TAM ( Davis, 1989) and TPB ( Ajzen, 1991) in technology adoption, the integration of Trust as a key factor offers a more comprehensive framework for understanding digital payment behavior. The results confirm that Trust is not only an antecedent of PU but also directly influences ATT, SN, and PBC.

This study’s empirical findings validate the C-TAM-TPB model (Combined TAM-TPB with Trust) by demonstrating that Trust plays a dual role in digital payment adoption. First, Trust enhances PU (β = 0.478, p < 0.001), indicating that users who perceive digital payments as secure and reliable also find them more beneficial. Second, Trust has a direct positive impact on ATT (β = 0.751, p < 0.001) and SN (β = 0.362, p < 0.001), confirming that when users trust digital payment providers, they develop a more positive perception of the technology and are more influenced by social endorsement. These relationships highlight that Trust acts as a bridge between technological perceptions (TAM) and behavioral intentions (TPB), addressing a critical gap in prior research ( Gefen et al., 2003; McKnight et al., 2002).

A particularly noteworthy finding of this study is the negative relationship between PU and ATT (β = -0.131, p = 0.024). This contradicts traditional TAM assumptions ( Wu & Chen, 2005) and suggests that, in Vietnam’s Northern mountainous regions, users may recognize the benefits of digital payments but still resist adoption due to ingrained cash-based habits. This finding underscores the importance of Trust as a mediating factor, as higher levels of trust in digital payments can mitigate resistance even when usefulness alone does not generate positive attitudes.

Moreover, this study extends the application of knowledge-sharing theories to digital payment adoption. Trust is a critical element in the diffusion of new technologies, as it reduces uncertainty and encourages users to rely on social proof before making adoption decisions ( Chow & Chan, 2008; Wang & Noe, 2010). This suggests that digital payment behavior is not just influenced by individual decision-making but also by social endorsement and peer-driven knowledge exchange.

By integrating Trust into TAM and TPB, this study advances existing technology acceptance theories in financial technology adoption. The C-TAM-TPB model provides a more holistic perspective, recognizing that digital payment adoption is not solely driven by ease of use and usefulness but is equally influenced by trust, social expectations, and perceived behavioral control. This aligns with recent research emphasizing the increasing role of security, privacy, and trust in financial technology adoption ( Giang et al., 2024; Siagian et al., 2022). The theoretical implications of this study extend beyond digital payments and offer valuable insights into broader fintech adoption, mobile banking, and e-wallet services. As emerging markets continue to transition from cash-based to digital economies, trust-centered frameworks will become increasingly important in explaining user behavior toward digital financial services.

### 5.3 Practical implications

While digital payments are primarily enabled by Internet and communication technologies, this study highlights the importance of addressing both technological and trust-related issues to enhance citizens’ decision to use these services. The Technology Acceptance Model (TAM) identifies perceived usefulness (PU) and perceived ease of use (PEU) alongside trust as critical factors influencing behavioral intention. Each factor significantly affects adoption through mediators such as attitude, perceived behavioral control, and subjective norms.

Careful attention must be given to both technological design and trust components to encourage citizens to adopt digital payments. Additionally, as noted earlier, novice users tend to prioritize trust in non-technological aspects over PEU and usefulness when forming their attitudes. This suggests that trust is more critical in shaping a user’s attitude toward digital payments than the technology’s perceived ease of use or usefulness. Major trust concerns include privacy protection, accuracy of information, and unauthorized access, among others. Trust-building mechanisms, such as transparent data privacy policies, fraud prevention systems, and community-driven endorsements, can help accelerate digital payment adoption. Policymakers should implement regulations that ensure consumer rights are protected in digital transactions, reinforcing public confidence in fintech solutions.

Fundamentally, trust has been empirically identified as a precursor to perceived usefulness (PU), which in turn influences attitude. This has important practical implications for improving attitudes toward digital payments. Providers of digital payment services should focus on developing trust-building mechanisms to attract novice users. Examples of such mechanisms include guarantees, increased familiarity through advertising, reliable customer service, and incentives for usage.

Additionally, leveraging peer-driven adoption strategies can be highly effective. Encouraging early adopters to act as digital payment ambassadors within their communities could create a cascading trust effect, increasing acceptance among hesitant users. Service providers should integrate referral programs and social proof elements into their marketing strategies to leverage the influence of subjective norms on digital payment adoption.

Once trust is established, enhancing the perceived usefulness of digital payments becomes crucial for attracting new users. This requires careful design aligning with users’ needs to communicate the service’s benefits effectively. Without initially addressing trust, even a well-designed digital payment system with high perceived usefulness may struggle to engage novice users.

Practically, this suggests that policymakers and service providers should implement trust-enhancing strategies, such as fraud prevention measures, peer-driven digital literacy campaigns, and transparency in financial transactions. Given that knowledge-sharing networks play a role in adoption, leveraging community-based programs to increase trust and familiarity with digital payments could prove highly effective in cash-dependent regions.

### 5.4 Limitations

While this study enhances the existing literature by integrating multiple models to examine users’ adoption of digital payments, it is essential to acknowledge its limitations. First, we used perceived usefulness to capture the overall benefits of digital payments for users. However, these benefits should be distinguished between economic and non-economic aspects, such as user satisfaction and service quality. Second, the number of farmers with low levels of education is less than that of others working away from home and commune-level civil servants. Farmers with low levels of education may be less likely to have adequate information to trust and adopt digital payment methods than cash. Therefore, future research should explore the impact of adopting digital payments on farmer income. It also should explore the role of trust in different financial ecosystems and investigate how digital literacy influences adoption. Longitudinal studies could provide further insights into how user trust and digital payment behavior evolve over time in emerging markets. Finally, qualitative research could provide deeper insights into the psychological and social barriers that prevent consumers from adopting digital payments. Conducting interviews or focus groups with users hesitant to adopt digital payment systems could help identify specific pain points and guide targeted intervention strategies.

## 6. Conclusion

The aim of this research is to propose an extended model that integrates Trust and the Technology Acceptance Model (TAM) with the Theory of Planned Behavior (TPB) to predict user adoption of digital payment methods more comprehensively. An extensive survey of digital payment users was conducted to test this research model empirically. As previously discussed, several new findings emerged regarding the roles of Trust, TAM, and TPB in digital payment adoption. The empirical results from the SEM indicate that all standardized path coefficients are positively significant, with the exception of the path from perceived usefulness to attitude. These findings carry significant implications for both practitioners and researchers.

A particularly noteworthy finding of this study is the negative relationship between PU and ATT. This contradicts traditional TAM assumptions ( Wu & Chen, 2005) and suggests that, in Vietnam’s Northern mountainous regions, users may recognize the benefits of digital payments but still resist adoption due to ingrained cash-based habits. This finding underscores the importance of Trust as a mediating factor, as higher levels of trust in digital payments can mitigate resistance even when usefulness alone does not generate positive attitudes.

Furthermore, this study provides valuable insights into the integration of knowledge-sharing frameworks within digital payment adoption. Trust is not just a factor influencing user acceptance but also plays a role in how information about digital payments is shared within communities. This suggests that adoption strategies should not only focus on improving system usability but also on fostering trust through social influence and community-based interventions.

From a theoretical perspective, this study refines the Trust-TAM-TPB model, positioning trust as a bridging factor between technology acceptance and behavioral intention. This integration offers a more comprehensive framework for understanding digital payment behavior in regions with low fintech penetration.

Practically, these findings suggest that financial institutions, policymakers, and technology developers should prioritize trust-building strategies. Strengthening fraud protection measures, launching targeted financial literacy programs, and leveraging peer recommendations could accelerate adoption. Service providers must not only improve system usability but also foster trust through transparent policies, consumer protection mechanisms, and community engagement initiatives.

Finally, the study highlights the need for further research to explore the dynamic nature of trust in digital payments. Given the rapid evolution of financial technology, longitudinal studies could provide deeper insights into how user perceptions shift over time, allowing policymakers and service providers to adjust their strategies accordingly.

## Ethics and consent

There was no ethics or institutional committee at Phenikaa University when this study was conducted. Our university published Decision No. 498/QĐ-ĐHP-KHCN of the Rector on promulgating the Regulations on ethical standards and integrity in scientific and technological activities of Phenikaa University on 30 ^th^ October 2020. It is a rule for all researchers and lecturers to create a healthy and professional research environment, ensuring standards and integrity in scientific research and in accordance with international practices. Our university has a medical ethics committee for the health sciences, but this committee has not yet been established for the social sciences and humanities. We have also recommended the establishment of a research ethics committee for our leaders.

In our research, we follow the best ethical practices. All respondents were asked for their permission, and the details of the questionnaires were explained before answering. The survey form was designed based on previous studies and checked carefully by colleagues and experts. The questions focus on two main parts: personal information and respondents’ perceptions of digital payments. The collected data is only used for the authors’ research purposes. Therefore, this study is low-risk in nature.

Informed consent for participation was obtained through written forms. Respondents were asked to provide personal information, including their name, address, age, education level, income, and occupation, which the interviewers recorded on the survey forms. Finally, participants reviewed their answers for accuracy and signed both the survey form and a receipt for the gift. We collected and stored written consent from all the participants.

## Author contributions

Truong Tuan Linh suggested ideas and wrote the “Introduction,” “Theoretical background”, “Methods” and “Conclusion” sections. Nguyen Thi Thanh Huyen wrote the “Empirical results” and “Discussion” sections.

## Data Availability

Reshare: Analysis of Factors Influencing the Use of Electronic Payment Methods in Consumer Behavior Among Households in the Northern Mountainous Region, Vietnam. https://reshare.ukdataservice.ac.uk/857466/. DOI: 10.5255/UKDA-SN-857466 The project contains the following underlying data:
- An Extension of Trust and TAM Model with TPB in the Adoption of Digital Payment.xlsx - Readme_An Extension of Trust and TAM Model with TPB in the Adoption of Digital Paymen.docx An Extension of Trust and Technology Acceptance Model with Theory of Planned Behavior in the Adoption of Digital Payment: An Empirical Study in Vietnam, 2023-2024 Data are available under the terms of the Creative Commons Attribution 4.0 International license (CC-BY 4.0). Reshare: Analysis of Factors Influencing the Use of Electronic Payment Methods in Consumer Behavior Among Households in the Northern Mountainous Region, Vietnam. https://reshare.ukdataservice.ac.uk/857466/ DOI: 10.5255/UKDA-SN-857466 The project contains the following extended data:
- Consent form_An Extension of Trust and TAM Model with TPB in the Adoption of Digital Payment_English.docx - Data dictionary_An Extension of Trust and TAM Model with TPB in the Adoption of Digital Payment.xlsx - Survey Guidelines.docx - Summary table of survey information.docx Data are available under the terms of the Creative Commons Attribution 4.0 International license (CC-BY 4.0).

## Software availability statement

In our study, SEM analysis was conducted using JASP, an open-source software.

• Source code available from: https://github.com/jasp-stats
• Archived software available from: https://jasp-stats.org/download/
• License: OSI approved open license software is under GNU GPLv2 (ASP-Engine) and GNU Affero GPL v3 (JASP-Desktop)

For readers seeking alternatives, we also recommend R with the lavaan package, which is a widely used tool for SEM and is also open access.
