# Supplementary material for: An extension of Trust and TAM model with TPB in the adoption of digital payment: An empirical study in Vietnam
Source: F1000Res. 2025 Apr 14;14:127. Originally published 2025 Jan 27. [Version 3] doi: 10.12688/f1000research.157763.3 (PMC11969139; doi:10.12688/f1000research.157763.3)
Supplement: Peer review history

# Reviewer report: Reviewer response for version 3

**DOI:** 10.5256/f1000research.180262.r377857

**Published:** 2026-01-08

**Authors:** Mohammad Rakibul Islam Bhuiyan

**Affiliations:** 1 Begum Rokeya University, Rangpur, Bangladesh

Based on the responses of the comment, I am satisfied and I recommend to index it now. Thanks for this opportunity to review this interesting work. Paper is considered for final indexing

Is the work clearly and accurately presented and does it cite the current literature?

No

If applicable, is the statistical analysis and its interpretation appropriate?

Partly

Are all the source data underlying the results available to ensure full reproducibility?

Yes

Is the study design appropriate and is the work technically sound?

Yes

Are the conclusions drawn adequately supported by the results?

Yes

Are sufficient details of methods and analysis provided to allow replication by others?

Partly

Reviewer Expertise:

Cashless Society, Digital Transformation, Industry 4.0, Emerging Technologies

I confirm that I have read this submission and believe that I have an appropriate level of expertise to confirm that it is of an acceptable scientific standard.

## References

[1] : An extension of Trust and TAM model with TPB in the adoption of digital payment: An empirical study in Vietnam. *F1000Research* .2025;14: 10.12688/f1000research.157763.3 10.12688/f1000research.157763.3 40191151 PMC11969139

# Reviewer report: Reviewer response for version 3

**DOI:** 10.5256/f1000research.180262.r377856

**Published:** 2025-04-22

**Authors:** Shenglin Ma

**Affiliations:** 1 North University of China, Taiyuan, Shanxi, China

The author has made excellent revisions. I have no further comments.

Is the work clearly and accurately presented and does it cite the current literature?

Yes

If applicable, is the statistical analysis and its interpretation appropriate?

Yes

Are all the source data underlying the results available to ensure full reproducibility?

Yes

Is the study design appropriate and is the work technically sound?

Yes

Are the conclusions drawn adequately supported by the results?

Yes

Are sufficient details of methods and analysis provided to allow replication by others?

Yes

Reviewer Expertise:

Cross border e-commerce； ESG;  fiance economics；construction economics；digital economics

I confirm that I have read this submission and believe that I have an appropriate level of expertise to confirm that it is of an acceptable scientific standard.

# Reviewer report: Reviewer response for version 2

**DOI:** 10.5256/f1000research.178866.r374193

**Published:** 2025-04-03

**Authors:** Shenglin Ma

**Affiliations:** 1 North University of China, Taiyuan, Shanxi, China

This article explores the research on digital payment applications based on the TAM model of trust in Vietnam. Generally, the content is complete, but the following modifications are needed.

 The author has done a good job , but needs to make changes in the following sections

 1. The abstract of the article is too much, please reduce it appropriately to the required number of words.

 2. Keywords should preferably be 3-5.

 3. The resolution of the color images of the article is guaranteed to be above 300 dpi.

 4. Ensure that the tables of the article are in a three-line table with a uniform format.

 5. Reference format should be unified into APA format to ensure that the literature has not been withdrawn.

Is the work clearly and accurately presented and does it cite the current literature?

Yes

If applicable, is the statistical analysis and its interpretation appropriate?

Yes

Are all the source data underlying the results available to ensure full reproducibility?

Yes

Is the study design appropriate and is the work technically sound?

Yes

Are the conclusions drawn adequately supported by the results?

Yes

Are sufficient details of methods and analysis provided to allow replication by others?

Yes

Reviewer Expertise:

Cross border e-commerce； ESG;  fiance economics；construction economics；digital economics

I confirm that I have read this submission and believe that I have an appropriate level of expertise to confirm that it is of an acceptable scientific standard, however I have significant reservations, as outlined above.

## References

[1] : The impact of economic policy uncertainty on the domestic value added rate of construction enterprise exports—evidence from China. *Journal of Asian Architecture and Building Engineering* .2025; 10.1080/13467581.2025.2479512 1-15 10.1080/13467581.2025.2479512

[2] : The influence of internet celebrity anchors’ reputation on consumers’ purchase intention in the context of digital economy: from the perspective of consumers’ initial trust. *Applied Economics* .2024;56(60) : 10.1080/00036846.2023.2299266 9189-9210 10.1080/00036846.2023.2299266

[3] : Tail risk contagion and multiscale spillovers in the green finance index and large US technology stocks. *International Review of Financial Analysis* .2025;97: 10.1016/j.irfa.2024.103865 10.1016/j.irfa.2024.103865

# Response

**Published:** 2025-04-04

**Authors:** Truong Tuan Linh

**Affiliations:** Business and Economics, Phenikaa University, Hanoi, Hanoi, Vietnam

**Dear Reviewer,**

****We sincerely appreciate the valuable time and effort you have taken to review our manuscript. Your insightful comments and constructive suggestions have been instrumental in helping us improve the quality and clarity of our paper.

             We have carefully considered each of your points and made the corresponding revisions to our manuscript. Please find below our point-by-point responses to your comments:

**Comment 1:  ** The abstract of the article is too much, please reduce it appropriately to the required number of words.

**Answer 1:**

             Thank you for your helpful comment. We have carefully revised and shortened the abstract to meet the journal’s word count guidelines while preserving the key objectives, methodology, findings, and practical implications of the study.

             Specifically, we have removed or condensed the following parts:

             1) Removed from Background section:“providing a comprehensive framework to analyze digital payment adoption in emerging markets.”

**Reason:** This phrase repeated what was already implied and was not essential for conveying the novelty of the model.

             2) Removed from Results section: “These insights challenge traditional TAM assumptions and emphasize the importance of trust in driving adoption.”

**Reason:** Although insightful, this interpretation was already reflected in the previous sentence and was removed to avoid redundancy.

             As a result, the revised abstract now adheres to word count guidelines, remains focused, and better emphasizes the study's key contributions.

             We appreciate your feedback that helped us improve the clarity and precision of the abstract.

**Comment** **2.** Keywords should preferably be 3-5.

**Answer 2:**

             Thank you very much for your recommendation regarding the number of keywords. We fully acknowledge the importance of keeping keywords concise and focused. After careful consideration, we have reviewed our current list and found that the five keywords selected are already the most appropriate and representative of the study’s content:

 Current Keywords: *acceptance behaviors, C-TAM-TPB, digital payment, digital economy, trust-related issues*

             These keywords effectively reflect the core components of our research, including the theoretical framework (C-TAM-TPB), the technological context (digital payment and digital economy), and the key influencing factor (trust).

             Therefore, while we sincerely appreciate your suggestion, we respectfully propose to retain the current five keywords as they best capture the scope and focus of the manuscript.

**Comment** **3.** The resolution of the color images of the article is guaranteed to be above 300 dpi.

**Answer 3:**

             Thank you for your comment. We have carefully reviewed the images included in the manuscript. We confirm that all figures have been processed using professional image editing software (Adobe Photoshop), and their resolution has been adjusted to meet the publication requirement of at least 300 dpi.

             We appreciate your attention to technical quality, and we have taken the necessary steps to ensure the images meet the journal’s standards.

**Comment** **4**. Ensure that the tables of the article are in a three-line table with a uniform format.

**Answer 4:**

             Thank you for your suggestion. We have reviewed all tables in the manuscript and confirm that they have been formatted according to the three-line table style as required. Specifically, each table now includes a top line, a line below the column headings, and a bottom line for clarity and uniform presentation.

             We appreciate your attention to formatting details, and we have ensured consistency across all tables in the article.

**Comment** **5**. Reference format should be unified into APA format to ensure that the literature has not been withdrawn.

**Answer 5:**

             Thank you for your valuable comment. We have thoroughly reviewed the entire reference list and ensured that all entries strictly follow the APA citation style, both in the main text and in the reference section. Additionally, we have verified that all cited sources are valid and none have been withdrawn.

             Furthermore, we have incorporated the three suggested references (Wen et al., 2024; Wu et al., 2025; Zeng et al., 2025) into the manuscript appropriately and formatted them consistently according to the APA style.

             We appreciate your careful attention to referencing accuracy and formatting, and we have made sure our manuscript meets the journal’s standards.

# Reviewer report: Reviewer response for version 2

**DOI:** 10.5256/f1000research.178866.r370430

**Published:** 2025-03-12

**Authors:** Mohammad Rakibul Islam Bhuiyan

**Affiliations:** 1 Begum Rokeya University, Rangpur, Bangladesh

Thank you for providing me with the opportunity to review the revised version of the article titled *"An extension of Trust and TAM model with TPB in the adoption of digital payment: An empirical study in Vietnam"* by Linh TT and Huyen NTT.

 After carefully reviewing the revised manuscript, I can confirm that the authors have made significant improvements, addressing most of the concerns I raised in my initial report.

 Below, I summarize my updated thoughts on the revisions:

 1. **Restructured Introduction and Abstract**

 The revised introduction and abstract effectively highlight the originality of the study and provide a clear and concise overview of the research objectives, methodology, and findings. The restructuring has enhanced the readability and focus of these sections, ensuring the key aspects of the study are well presented.

 2. **Updated Literature Review**

 The authors have incorporated more recent references, particularly from 2022 to 2025, which strengthens the theoretical foundation of the study. This update improves the relevance of the study in the context of current research trends in digital payment adoption, particularly in emerging economies like Vietnam.

 3. **Expanded Methodology Section**

 The methodology section has been significantly enhanced by providing additional details on the sampling procedure, the justification of the sample size, and the validation of the survey instrument. These improvements greatly enhance the transparency and rigor of the research design.

 4. **Clarified Statistical Analysis**

 The explanation of the SEM approach is now clearer, with a more detailed description of model fit indices and the relationship between statistical findings and research hypotheses. This provides greater clarity on the statistical methods used, improving the overall methodological transparency.

 5. **Strengthened Theoretical Contributions**

 The authors have done a commendable job of elaborating on how the integration of Trust, TAM, and TPB contributes to advancing existing technology acceptance models. The positioning of digital payment adoption as a knowledge-sharing process in emerging economies adds a novel theoretical perspective to the field.

 6. **Expanded Discussion and Practical Implications**

 The authors have strengthened the discussion and practical implications by offering deeper interpretations of the findings. The inclusion of specific recommendations for policymakers, financial institutions, and technology providers enhances the real-world relevance of the study.

 7. **Added Limitations and Future Research Section**

 The inclusion of a limitations section, which addresses geographical constraints and methodological considerations, adds a valuable layer of transparency. The authors' proposals for future research directions are insightful, particularly the suggestions for cross-country validation, longitudinal studies, and the inclusion of additional psychological constructs.

 8. **Updated References Section**

 The reference list has been updated to include the latest studies and to ensure proper citation formatting, which enhances the overall academic rigor of the paper.

 Conclusion

 The authors have effectively addressed the revisions and substantially improved the manuscript. The updated version demonstrates significant improvements in clarity, depth, and theoretical contributions. I am now confident in endorsing the manuscript for indexed with no further reservations.

Is the work clearly and accurately presented and does it cite the current literature?

No

If applicable, is the statistical analysis and its interpretation appropriate?

Partly

Are all the source data underlying the results available to ensure full reproducibility?

Yes

Is the study design appropriate and is the work technically sound?

Yes

Are the conclusions drawn adequately supported by the results?

Yes

Are sufficient details of methods and analysis provided to allow replication by others?

Partly

Reviewer Expertise:

Cashless Society, Digital Transformation, Industry 4.0, Emerging Technologies

I confirm that I have read this submission and believe that I have an appropriate level of expertise to confirm that it is of an acceptable scientific standard.

## References

[1] : An extension of Trust and TAM model with TPB in the adoption of digital payment: An empirical study in Vietnam. *F1000Research* .2025;14: 10.12688/f1000research.157763.2 10.12688/f1000research.157763.2 40191151 PMC11969139

# Reviewer report: Reviewer response for version 1

**DOI:** 10.5256/f1000research.173261.r363009

**Published:** 2025-02-27

**Authors:** Dmaithan Almajali

**Affiliations:** 1 Applied Science Private University, Amman, Amman Governorate, Jordan

The paper presents a limited and not adequate methodological and practical assessment. Also the theoretical foundations seem to be not completely.

 The paper requests a strong revision by the authors in order to improve the quality of the paper for future submission.

 Briefly I suggest as following some comments on how, on my opinion, the theoretical background, as well as practical development can be strengthened by detailing some and adding quality to this manuscript.

 The introduction part can benefit from being restructured a little bit as well as the abstract by emphasizing the aspects of originality for this inquiry and the methods as well.

 The risk of leaving the introduction as it is, is to appear low clear in the readers' mind as for the aim of the paper.

 However, it should be advisable to implement with prior works published on international peer review journals in order to strengthen the literature and to oriented this topic to a particular stream of research on knowledge sharing.

 While the premises of the study appear original, the discussion of conclusions and implications should be enriched. You can elaborate more reframing the section and comment accordingly your results to the implications that emerged as relevant through your study in theory and practice. Also limitations and future perspectives could be added.

Is the work clearly and accurately presented and does it cite the current literature?

Partly

If applicable, is the statistical analysis and its interpretation appropriate?

Yes

Are all the source data underlying the results available to ensure full reproducibility?

Yes

Is the study design appropriate and is the work technically sound?

No

Are the conclusions drawn adequately supported by the results?

No

Are sufficient details of methods and analysis provided to allow replication by others?

No

Reviewer Expertise:

ERP, CRM, cryptocurency,

I confirm that I have read this submission and believe that I have an appropriate level of expertise to confirm that it is of an acceptable scientific standard, however I have significant reservations, as outlined above.

# Response

**Published:** 2025-03-01

**Authors:** Truong Tuan Linh

**Affiliations:** Business and Economics, Phenikaa University, Hanoi, Hanoi, Vietnam

Dear Dr Dmaithan Almajali,

 We sincerely appreciate the constructive feedback provided by Reviewer. The insightful comments have guided us in improving the clarity, depth, and contributions of our manuscript. Based on your recommendations, we have made the following revisions:

 - Restructured the Introduction and Abstract: We have refined these sections to emphasize the originality of our study and clearly present our methodology and contributions.

 - Strengthened Literature Review: We have incorporated additional references from international peer-reviewed journals to align our study with current research trends and knowledge-sharing perspectives.

 - Expanded Discussion and Implications: We have enriched the discussion by elaborating on how our findings contribute to theoretical and practical implications. Our revisions ensure a clearer connection between our results and their significance in digital payment adoption.

 - Added a Limitations and Future Research Section: To enhance the study’s rigor, we have explicitly addressed the limitations of our research and proposed directions for future studies, including cross-country validation, longitudinal analysis, and additional psychological constructs.

 We are grateful for the reviewer’s valuable suggestions and believe these revisions have significantly improved our manuscript. Thank you for your time and effort in reviewing our work.

**Comment 1: ** The introduction part can benefit from being restructured a little bit as well as the abstract by emphasizing the aspects of originality for this inquiry and the methods as well.

 The risk of leaving the introduction as it is, is to appear low clear in the readers' mind as for the aim of the paper

**Answer 1:**

 1.1. We revised the abstract to highlight the uniqueness of this research and clearly define the methodology used.

**Background**

 Digital payment systems are pivotal in the digital economy, relying on the interplay between internet technology and e-vendors. While the Technology Acceptance Model (TAM) and the Theory of Planned Behavior (TPB) have been extensively used to explain technology adoption, the role of trust in financial technology adoption remains underexplored. This study addresses this gap by developing an extended Trust-TAM-TPB model, providing a comprehensive framework to analyze digital payment adoption in emerging markets.

**Methods**

 A quantitative approach was adopted, analyzing survey data from 509 respondents using Structural Equation Modeling (SEM). The extended model examines both technological factors (perceived usefulness, perceived ease of use) and trust-related factors (trust’s influence on behavioral intention via subjective norms, attitude, and perceived behavioral control).

**Results**

 Findings confirm that trust is a significant determinant of digital payment adoption, influencing both perceived usefulness and subjective norms. However, a negative relationship was found between perceived usefulness (PU) and attitude (ATT) (β = -0.131, p = 0.024), suggesting that while users recognize the benefits of digital payments, their attitudes may still be shaped by traditional cash-based habits and security concerns. These insights challenge traditional TAM assumptions and emphasize the importance of trust in driving adoption.

**Conclusions**

 This study contributes to technology acceptance literature by integrating trust into the TAM-TPB framework and highlighting its dual role in shaping both perceived usefulness and behavioral intention. Practically, the findings suggest that policymakers and financial institutions should prioritize trust-building strategies, including fraud prevention measures, financial literacy programs, and transparent transaction policies, to accelerate digital payment adoption in developing economies. These insights are particularly relevant for Vietnam’s Northern mountainous regions, where digital payment penetration remains low.

 1.2. We restructured the introduction to improve clarity and emphasize the originality and contribution of the study.

 Digital payments have transformed financial transactions, particularly in the wake of technological advancements and changing consumer behaviors. The increasing reliance on digital payment methods has sparked significant academic interest, particularly regarding the factors influencing user adoption. While the TAM and TPB have been widely applied to explain technology acceptance, emerging research highlights the critical role of trust in financial technology adoption (Gefen et al., 2003; McKnight et al., 2002). However, limited research has examined how trust integrates with TAM and TPB to shape digital payment adoption, particularly in regions with low digital financial literacy and persistent cash-based economies. This study seeks to address this gap by developing an extended model Trust-TAM-TPB to examine digital payment adoption in Vietnam’s Northern mountainous provinces. This context presents a unique case, as digital payment adoption remains relatively low due to trust concerns, infrastructure limitations, and entrenched consumer habits. By analyzing responses from 509 users through Structural Equation Modeling (SEM), this research aims to contribute both theoretically and practically by providing new insights into the role of trust in shaping digital financial behavior. The study findings offer implications for financial institutions, policymakers, and technology providers aiming to accelerate digital payment adoption in developing markets. The rest of the paper is structured as follows: Section 2 provides theoretical background. Section 3 outlines the methods. Section 4 presents empirical results. Section 5 focuses on discussing the main results and suggesting some implications. Finally, the authors conclude some limitations and raise potential areas for future research in Section 6.

**Comment 2:  ** However, it should be advisable to implement with prior works published on international peer review journals in order to strengthen the literature and to oriented this topic to a particular stream of research on knowledge sharing.

 While the premises of the study appear original, the discussion of conclusions and implications should be enriched. You can elaborate more reframing the section and comment accordingly your results to the implications that emerged as relevant through your study in theory and practice. Also limitations and future perspectives could be added.

**Answer 2: ** We expanded the discussion section, providing a deeper interpretation of results and linking findings to Discusion and Conclusions parts as follows:

***5.1. Discussion results***

**TAM model**

 Our findings align with prior literature on technology adoption and trust, reinforcing the idea that perceived ease of use (PEU) and trust (TR) are critical for overcoming digital payment resistance (Gefen et al., 2003; McKnight et al., 2002). The negative PU → ATT relationship observed in this study suggests that usefulness alone is not sufficient in encouraging adoption, especially in economies with entrenched cash-based behaviors. This aligns with knowledge-sharing theories, where social influence and trust networks play a crucial role in facilitating the adoption of new technologies (Chow & Chan, 2008; Wang & Noe, 2010).

**TPB model**

 Findings related to subjective norms (SN) further support the knowledge-sharing perspective, as social influence plays a key role in shaping user trust in digital payments. Reliable information from trusted sources like friends, family, or communities boosts confidence, reinforcing prior research that suggests trust-based peer recommendations are essential for technology adoption (Bock et al., 2005; Lin, 2007).

**Trust and TAM -TPB relationship**

 This study extends the literature by showing that trust acts as a bridge between technological perceptions (TAM) and behavioral intentions (TPB), further reinforcing the interconnected role of technology and trust in financial decision-making. The dual role of trust in shaping both perceived usefulness (PU) and behavioral control aligns with broader fintech research, emphasizing that trust is a prerequisite for digital financial engagement (Nguyen & Ao, 2022; Siagian et al., 2022).

***5.2. Theoretical contributions***

 In line with this existing research, the findings of this study provide significant theoretical contributions by extending the TAM and TPB with Trust in the context of digital payment adoption. While previous studies have separately examined TAM (Davis, 1989) and TPB (Ajzen, 1991) in technology adoption, the integration of Trust as a key factor offers a more comprehensive framework for understanding digital payment behavior. The results confirm that Trust is not only an antecedent of PU but also directly influences ATT, SN, and PBC.

 This study’s empirical findings validate the C-TAM-TPB model (Combined TAM-TPB with Trust) by demonstrating that Trust plays a dual role in digital payment adoption. First, Trust enhances PU (β = 0.478, p < 0.001), indicating that users who perceive digital payments as secure and reliable also find them more beneficial. Second, Trust has a direct positive impact on ATT (β = 0.751, p < 0.001) and SN (β = 0.362, p < 0.001), confirming that when users trust digital payment providers, they develop a more positive perception of the technology and are more influenced by social endorsement. These relationships highlight that Trust acts as a bridge between technological perceptions (TAM) and behavioral intentions (TPB), addressing a critical gap in prior research (Gefen et al., 2003; McKnight et al., 2002).

 A particularly noteworthy finding of this study is the negative relationship between PU and ATT (β = -0.131, p = 0.024). This contradicts traditional TAM assumptions (Wu & Chen, 2005) and suggests that, in Vietnam’s northern mountainous regions, users may recognize the benefits of digital payments but still resist adoption due to ingrained cash-based habits. This finding underscores the importance of Trust as a mediating factor, as higher levels of trust in digital payments can mitigate resistance even when usefulness alone does not generate positive attitudes.

 Moreover, this study extends the application of knowledge-sharing theories to digital payment adoption. Trust is a critical element in the diffusion of new technologies, as it reduces uncertainty and encourages users to rely on social proof before making adoption decisions (Chow & Chan, 2008; Wang & Noe, 2010). This suggests that digital payment behavior is not just influenced by individual decision-making but also by social endorsement and peer-driven knowledge exchange.

 By integrating Trust into TAM and TPB, this study advances existing technology acceptance theories in financial technology adoption. The C-TAM-TPB model provides a more holistic perspective, recognizing that digital payment adoption is not solely driven by ease of use and usefulness but is equally influenced by trust, social expectations, and perceived behavioral control. This aligns with recent research emphasizing the increasing role of security, privacy, and trust in financial technology adoption (Giang et al., 2024; Siagian et al., 2022). The theoretical implications of this study extend beyond digital payments and offer valuable insights into broader fintech adoption, mobile banking, and e-wallet services. As emerging markets continue to transition from cash-based to digital economies, trust-centered frameworks will become increasingly important in explaining user behavior toward digital financial services.

***5.3. Practical implications***

 Trust-building mechanisms, such as transparent data privacy policies, fraud prevention systems, and community-driven endorsements, can help accelerate digital payment adoption. Policymakers should implement regulations that ensure consumer rights are protected in digital transactions, reinforcing public confidence in fintech solutions.

 Additionally, leveraging peer-driven adoption strategies can be highly effective. Encouraging early adopters to act as digital payment ambassadors within their communities could create a cascading trust effect, increasing acceptance among hesitant users. Service providers should integrate referral programs and social proof elements into their marketing strategies to leverage the influence of subjective norms on digital payment adoption.

 Practically, this suggests that policymakers and service providers should implement trust-enhancing strategies, such as fraud prevention measures, peer-driven digital literacy campaigns, and transparency in financial transactions. Given that knowledge-sharing networks play a role in adoption, leveraging community-based programs to increase trust and familiarity with digital payments could prove highly effective in cash-dependent regions.

***5.4. Limitations***

 It also should explore the role of trust in different financial ecosystems and investigate how digital literacy influences adoption. Longitudinal studies could provide further insights into how user trust and digital payment behavior evolve over time in emerging markets. Finally, qualitative research could provide deeper insights into the psychological and social barriers that prevent consumers from adopting digital payments. Conducting interviews or focus groups with users hesitant to adopt digital payment systems could help identify specific pain points and guide targeted intervention strategies.

**6. Conclusion**

 A particularly noteworthy finding of this study is the negative relationship between PU and ATT . This contradicts traditional TAM assumptions (Wu & Chen, 2005) and suggests that, in Vietnam’s Northern mountainous regions, users may recognize the benefits of digital payments but still resist adoption due to ingrained cash-based habits. This finding underscores the importance of Trust as a mediating factor, as higher levels of trust in digital payments can mitigate resistance even when usefulness alone does not generate positive attitudes. Finally, qualitative research could provide deeper insights into the psychological and social barriers that prevent consumers from adopting digital payments. Conducting interviews or focus groups with users hesitant to adopt digital payment systems could help identify specific pain points and guide targeted intervention strategies.

 Furthermore, this study provides valuable insights into the integration of knowledge-sharing frameworks within digital payment adoption. Trust is not just a factor influencing user acceptance but also plays a role in how information about digital payments is shared within communities. This suggests that adoption strategies should not only focus on improving system usability but also on fostering trust through social influence and community-based interventions.

 From a theoretical perspective, this study refines the Trust-TAM-TPB model, positioning trust as a bridging factor between technology acceptance and behavioral intention. This integration offers a more comprehensive framework for understanding digital payment behavior in regions with low fintech penetration.

 Practically, these findings suggest that financial institutions, policymakers, and technology developers should prioritize trust-building strategies. Strengthening fraud protection measures, launching targeted financial literacy programs, and leveraging peer recommendations could accelerate adoption. Service providers must not only improve system usability but also foster trust through transparent policies, consumer protection mechanisms, and community engagement initiatives.

 Finally, the study highlights the need for further research to explore the dynamic nature of trust in digital payments. Given the rapid evolution of financial technology, longitudinal studies could provide deeper insights into how user perceptions shift over time, allowing policymakers and service providers to adjust their strategies accordingly.

 We really appreciate your help!

 Sincerely yours,

 Truong Tuan Linh

# Reviewer report: Reviewer response for version 1

**DOI:** 10.5256/f1000research.173261.r363010

**Published:** 2025-02-04

**Authors:** Mohammad Rakibul Islam Bhuiyan

**Affiliations:** 1 Begum Rokeya University, Rangpur, Bangladesh

**1. Overall Evaluation:**

 The paper titled *"An extension of Trust and TAM model with TPB in the adoption of digital payment: An empirical study in Vietnam"* presents a timely and relevant study on digital payment adoption, with a particular focus on Vietnam. The study contributes to the body of knowledge by integrating the Trust Model, the Technology Acceptance Model (TAM), and the Theory of Planned Behavior (TPB) in a comprehensive framework. However, there are several areas where revisions are needed to improve the clarity, depth, and completeness of the research.

**2. Clarity and Precision of the Presentation:**

 The paper is generally well-written but could benefit from further refinement in some sections, particularly in the literature review and the interpretation of results. The connection between the theoretical models (Trust, TAM, TPB) could be more clearly articulated, specifically how they complement each other in the context of digital payment adoption. The introduction could also be expanded to better emphasize the research gap and the study’s contribution to the field.

**3. Literature Review and Citations:**

 While the paper cites some foundational studies, it is noticeably lacking in recent references (2022-2025). The literature review should be updated to include more recent research on digital payment adoption, especially considering the rapidly evolving nature of the digital payment landscape. Relevant studies on the impact of trust, technology acceptance, and behavioral intentions in digital finance should be integrated to strengthen the paper’s theoretical foundation.

**4. Methodology and Analysis:**

 The methodology is generally sound, but more detail is needed to clarify certain aspects of the research design. For example, the sampling process and sample size justification should be provided more thoroughly. Additionally, the survey instrument used to collect data could benefit from a more detailed explanation of its validation process. The use of structural equation modeling (SEM) is appropriate, but the model could be more thoroughly explained, particularly with regard to the measurement model and the path coefficients.

**5. Statistical Analysis:**

 The statistical analysis is partly addressed, but there are areas for improvement. It would be helpful to provide more detailed explanations of how the validity and reliability of the constructs were assessed. Additionally, further discussion of the model fit indices and how they support the hypotheses would strengthen the analysis. A clearer discussion of the limitations of the model would also be beneficial.

**6. Contribution to the Field:**

 The paper makes a solid contribution to understanding digital payment adoption in Vietnam, but the proposed model’s novelty and value would be more apparent with some improvements in the literature review, methodology, and analysis sections. Once these issues are addressed, this paper could provide important insights into how trust, technology acceptance, and behavioral intentions influence digital payment adoption in emerging economies like Vietnam.

**7. Recommendations for Revision:**

- **Literature Update:** Add more recent citations (2022-2025) to reflect the latest developments in the digital payment domain.
- **Methodology:** Provide more details on the sampling procedure, sample size justification, and survey instrument validation.
- **Statistical Analysis:** Clarify the SEM approach, provide more details on model fit indices, and discuss how the statistical findings relate to the hypotheses.
- **Theoretical Framework:** Strengthen the explanation of how the integrated model (Trust, TAM, and TPB) contributes to the field of digital payment adoption, and clarify the relationships between the constructs.

Once the above points are addressed, this paper has the potential to contribute significantly to the literature on digital payment adoption, particularly in the context of developing countries like Vietnam.

Is the work clearly and accurately presented and does it cite the current literature?

No

If applicable, is the statistical analysis and its interpretation appropriate?

Partly

Are all the source data underlying the results available to ensure full reproducibility?

Yes

Is the study design appropriate and is the work technically sound?

Yes

Are the conclusions drawn adequately supported by the results?

Yes

Are sufficient details of methods and analysis provided to allow replication by others?

Partly

Reviewer Expertise:

Cashless Society, Digital Transformation, Industry 4.0, Emerging Technologies

I confirm that I have read this submission and believe that I have an appropriate level of expertise to confirm that it is of an acceptable scientific standard, however I have significant reservations, as outlined above.

# Response

**Published:** 2025-03-01

**Authors:** Truong Tuan Linh

**Affiliations:** Business and Economics, Phenikaa University, Hanoi, Hanoi, Vietnam

Dear Dr. Mohammad Rakibul Islam Bhuiyan,

 We sincerely appreciate the constructive feedback provided by the reviewer. Their insightful comments have helped us refine and enhance the quality of our manuscript. Based on their recommendations, we have made the following revisions:

 - **Literature Update:** We have incorporated recent studies (2022–2025) to reflect the latest developments in digital payment adoption, emphasizing the role of trust, security, and behavioral intention. These updates strengthen the theoretical foundation of our study and align it with current research trends.

 - **Methodology Enhancement:** We have provided a more detailed explanation of the sampling procedure, sample size justification, and survey instrument validation. This includes clarifying the stratified sampling approach, justifying the selection of 509 responses for SEM analysis, and outlining the three-step validation process (expert review, pilot study, and statistical validation).

 - **Statistical Analysis Clarification:** The SEM approach is now more explicitly detailed, with an improved explanation of the measurement and structural models. We have also expanded the discussion on model fit indices, linking them to established benchmarks. Additionally, we have clarified how our statistical findings relate to the hypotheses, particularly addressing unexpected results, such as the negative relationship between perceived usefulness and attitude.

 - **Theoretical Framework Enhancement:** We have strengthened the discussion in Section 5.2 (Theoretical Contributions) to emphasize how the integration of Trust, TAM, and TPB advances existing models of digital payment adoption. The revised section highlights the dual role of trust in influencing both perceived usefulness and social/behavioral factors, providing a more holistic framework for understanding digital payment behavior.

 We appreciate the reviewer’s valuable input and believe that these revisions significantly improve the manuscript’s clarity, rigor, and contribution to the field.

**Details are below:**

**Comment 1: Literature Update:** Add more recent citations (2022-2025) to reflect the latest developments in the digital payment domain.

**Answer 1:** To address this comment, I updated the Literature Review section under Theoretical Background to incorporate recent studies (2022-2025) that reflect the latest advancements in digital payment adoption, trust in fintech, and post-pandemic digital payment behavior:

 Recent studies have highlighted the rapid evolution of digital payment systems, particularly in response to increased digitalization and post-pandemic consumer behavior shifts. Siagian et al. (2022) investigated the role of trust and security concerns in digital payment adoption, emphasizing the interplay between technological ease of use and consumer confidence. Meanwhile, Ranpariya et al. (2023) applied the UTAUT model to analyze the impact of social influence and security perception on mobile payment adoption. Al-Qudah et al. (2024) further expanded on generational differences in digital payment adoption, identifying Generation Z's preference for contactless transactions driven by perceived convenience and security. Additionally, Giang et al. (2024) examined how consumer trust mediates the adoption of electronic payments in e-commerce settings. These recent insights reinforce the significance of trust, security, and perceived usefulness in digital payment adoption, further justifying this study's integration of the Trust-TAM-TPB model.

**Comment 2:** **Methodology:** Provide more details on the sampling procedure, sample size justification, and survey instrument validation.

**Answer 2: ** To address this comment, we expanded the methodology section with detailed explanations:

 This study employed a multi-stage stratified sampling approach to ensure diverse representation across different demographic segments. The research was conducted in Lang Son and Cao Bang provinces, which were selected due to their relatively low digital payment adoption rates. In the first stage, we identified key districts within each province where digital payments were increasingly promoted. In the second stage, a random sampling technique was used to select individual respondents who had prior exposure to digital payments. Eligibility criteria included: (1) individuals aged 18 and above, (2) those who had engaged in at least one digital transaction in the past six months. This approach ensured a mix of urban and rural respondents, capturing diverse perspectives on digital payment

 The sample size of 509 valid responses was determined based on guidelines for Structural Equation Modeling (SEM).Hair et al. (2019) recommend a minimum of 200 cases for SEM analysis, with a ratio of 10:1 per observed variable. Given that our model includes multiple constructs, a sample exceeding 500 was targeted to enhance statistical power and model stability. The sample size follows recommendations from Kline (2023), which suggest that larger samples improve the robustness of SEM path estimates. This ensures that factor loadings and path coefficients remain stable, reducing the risk of sampling bias.

 To ensure the accuracy and reliability of the survey instrument, a three-stage validation process was conducted before data collection. First, the questionnaire was developed based on established scales from previous studies on Technology Acceptance Model (TAM), Theory of Planned Behavior (TPB), and Trust in Digital Payments (Ajzen, 1991; Davis, 1989; Gefen et al., 2003). To assess content validity, the survey items were reviewed by three fintech experts and two academic researchers specializing in technology adoption and digital payments. Their feedback helped refine the wording and ensure the constructs accurately captured user perceptions in the Vietnamese context. Second, a pilot study was conducted with 100 participants from Thai Nguyen and Cao Bang to evaluate the clarity and comprehensibility of the questionnaire. Participants were asked to highlight any ambiguous or difficult-to-understand questions. Based on their feedback, minor wording adjustments were made to improve clarity without altering the intended meaning of the items. Finally, statistical validation of the instrument was planned before hypothesis testing. Specifically, Exploratory Factor Analysis (EFA) and Confirmatory Factor Analysis (CFA) would be employed to assess construct validity, while Cronbach’s alpha, Composite Reliability (CR), and Average Variance Extracted (AVE) would be used to evaluate internal consistency and reliability. The results of these tests are reported in the Results section. By implementing this rigorous validation process before the main data collection, the study ensured that the measurement instrument was robust and suitable for examining the proposed research model.

**Comment 3: Statistical Analysis:** Clarify the SEM approach, provide more details on model fit indices, and discuss how the statistical findings relate to the hypotheses.

**Answer 3: ** To enhance the clarity of the Structural Equation Modeling (SEM) approach, additional details are provided in *4.1. Measurement model and 4.2. Structural equation model *regarding the analytical procedures, model fit indices, and how the statistical findings relate to the proposed hypotheses.

***4.1. Measurement model***

 This study follows a two-step SEM approach as recommended by Hair et al. (2019). In the first step, the measurement model was assessed using Confirmatory Factor Analysis (CFA) to ensure construct validity, reliability, and overall model fit. Only after the measurement model demonstrated an acceptable fit was the structural model estimated. The second step involved testing the structural model, where maximum likelihood estimation (MLE) was applied to examine the relationships among latent variables. MLE was selected due to its robustness in handling non-normality and its effectiveness when the sample size exceeds 200, as supported by Kline (2018).

 Before proceeding with SEM model testing to evaluate the hypotheses, we conducted EFA and CFA to assess the variables' construct and confirm the reliability and validity of the measurement model. Table 2 shows that the total Cronbach’s alpha is 0.938 greater than 0.6, and all observed variables have Cronbach’s alpha coefficients ≥ 0.935. The results also indicate that seven variables were extracted from 30 observed factors; the eigenvalue value is 1.053 (>1) with a variance of 71.62%. The results of the EFA in Table 2 show that the factor loadings of the items ranged from 0.661 to 0.853, and the construct of items is consistent with the literature.  The test coefficient KMO = 0.916 satisfies the 0.5 < KMO< 1, showing that the exploratory factor analysis is appropriate for our data. The Chi-square statistic of the Bartlett test reached 9630.515, with the p-value = 0.000, showing that the data is suitable and reliable for performing the EFA method.

*****4.2. Structural equation model*****

**_10_**

****Comment 4:** **Theoretical Framework:****

**Answer 4:**

***5.2. Theoretical contributions***

 CFA allows for testing the validity and accuracy of specific models constructed based on data and theoretical foundations. We assess the goodness-of-fit of the measurement model using various tests, including the Chi-square test (χ2), Comparative fit index (CFI), Tucker–Lewis Index (TLI), and Root mean squared error of approximation (RMSEA), composite reliability (CR) and the average variance extracted (AVE). As shown in Table 3, the composite reliability (CR) of latent variables exceeds 0.836, and the AVE values range from 0.560 to 0.687.  In addition, several fit indices were analyzed. The chi-square statistic (χ²/df = 2.486) was within the acceptable threshold of less than 3, indicating a good fit  (Hair et al., 2019). The CFI = 0.943 and TLI = 0.931 were both above the recommended threshold of 0.90, confirming that the model exhibited strong comparative and incremental fit. Additionally, the RMSEA = 0.05 was below the upper limit of 0.08, signifying a satisfactory absolute fit. The SRMR = 0.03 was well below the 0.08 cutoff, further reinforcing the model’s robustness. Collectively, these indices demonstrate that the proposed model achieves an adequate fit, allowing for meaningful interpretation of the structural relationships. The results from the structural model estimation support most of the hypothesized relationships, confirming the theoretical foundations of the Trust-TAM-TPB model in the context of digital payment adoption. Notably, perceived ease of use (PEU) was found to have a significant positive impact on trust (β = 0.404, p < 0.001). This finding aligns with previous research (Gefen et al., 2003), which suggests that when users perceive a digital payment system as easy to use, they are more likely to develop confidence in its reliability and security. Additionally, trust was shown to significantly enhance perceived usefulness (PU) (β = 0.478, p < 0.001), reinforcing the notion that users find digital payment systems more beneficial when they have confidence in their security and operational effectiveness. A key theoretical confirmation from the findings is the strong influence of attitude (ATT) on digital payment adoption (DPA) (β = 0.186, p = 0.002). This result supports Hypothesis H and aligns with the Theory of Planned Behavior (TPB), where a positive attitude toward a technology significantly increases the likelihood of its adoption. Similarly, subjective norms (SN) and perceived behavioral control (PBC) were also found to be strong predictors of adoption, further reinforcing the relevance of TPB in digital payment contexts. However, an unexpected finding emerged regarding the relationship between perceived usefulness (PU) and attitude (ATT) (β = -0.131, p = 0.024). Contrary to previous research based on TAM (Wu & Chen, 2005), this result suggests that users in the studied regions may not necessarily develop a positive attitude toward digital payments even when they acknowledge their usefulness. A potential explanation is that long-standing cash-based habits in Vietnam’s Northern mountainous areas create resistance to shifting to digital transactions, despite recognizing their benefits. This insight suggests that beyond promoting usefulness, digital payment providers should emphasize trust-building strategies and behavioral incentives to encourage attitudinal change and subsequent adoption. Overall, the findings validate the core structure of the Trust-TAM-TPB model while revealing context-specific nuances that should be considered in digital payment adoption strategies. The integration of trust within the model highlights its essential role in shaping perceived usefulness and behavioral intentions, offering practical insights for financial institutions and policymakers aiming to increase digital payment penetration in emerging markets.  Strengthen the explanation of how the integrated model (Trust, TAM, and TPB) contributes to the field of digital payment adoption, and clarify the relationships between the constructs. We added in Section 5.2: Theoretical Contributions, as it builds upon the study's findings The previous body of literature concerning intentions to adopt digital payments has primarily relied on established theories such as TRA, TAM, TPB, UTAUT, and UTATU2 (Kabir et al., 2017; Susanto et al., 2022). In line with this existing research, the findings of this study provide significant theoretical contributions by extending the TAM and TPB with Trust in the context of digital payment adoption. While previous studies have separately examined TAM (Davis, 1989) and TPB (Ajzen, 1991) in technology adoption, the integration of Trust as a key factor offers a more comprehensive framework for understanding digital payment behavior. The results confirm that Trust is not only an antecedent of PU but also directly influences ATT, SN, and PBC. This study’s empirical findings validate the C-TAM-TPB model (Combined TAM-TPB with Trust) by demonstrating that Trust plays a dual role in digital payment adoption. First, Trust enhances PU (β = 0.478, p < 0.001), indicating that users who perceive digital payments as secure and reliable also find them more beneficial. Second, Trust has a direct positive impact on ATT (β = 0.751, p < 0.001) and SN (β = 0.362, p < 0.001), confirming that when users trust digital payment providers, they develop a more positive perception of the technology and are more influenced by social endorsement. These relationships highlight that Trust acts as a bridge between technological perceptions (TAM) and behavioral intentions (TPB), addressing a critical gap in prior research (Gefen et al., 2003; McKnight et al., 2002). A particularly noteworthy finding of this study is the negative relationship between PU and ATT (β = -0.131, p = 0.024). This contradicts traditional TAM assumptions (Wu & Chen, 2005) and suggests that, in Vietnam’s northern mountainous regions, users may recognize the benefits of digital payments but still resist adoption due to ingrained cash-based habits. This finding underscores the importance of Trust as a mediating factor, as higher levels of trust in digital payments can mitigate resistance even when usefulness alone does not generate positive attitudes. By integrating Trust into TAM and TPB, this study advances existing technology acceptance theories in financial technology adoption. The C-TAM-TPB model provides a more holistic perspective, recognizing that digital payment adoption is not solely driven by ease of use and usefulness but is equally influenced by trust, social expectations, and perceived behavioral control. This aligns with recent research emphasizing the increasing role of security, privacy, and trust in financial technology adoption (Giang et al., 2024; Siagian et al., 2022). The theoretical implications of this study extend beyond digital payments and offer valuable insights into broader fintech adoption, mobile banking, and e-wallet services. As emerging markets continue to transition from cash-based to digital economies, trust-centered frameworks will become increasingly important in explaining user behavior toward digital financial services. Thank you for your support! Sincerely, Truong Tuan Linh
